# A review on medical imaging synthesis using deep learning and its clinical applications

**DOI:** 10.1002/acm2.13121

**Published:** 2020-12-11

**Authors:** Tonghe Wang, Yang Lei, Yabo Fu, Jacob F. Wynne, Walter J. Curran, Tian Liu, Xiaofeng Yang

**Affiliations:** ^1^ Department of Radiation Oncology Emory University Atlanta GA USA; ^2^ Winship Cancer Institute Emory University Atlanta GA USA

**Keywords:** CT, deep learning, image synthesis, MRI, PET, radiation therapy

## Abstract

This paper reviewed the deep learning‐based studies for medical imaging synthesis and its clinical application. Specifically, we summarized the recent developments of deep learning‐based methods in inter‐ and intra‐modality image synthesis by listing and highlighting the proposed methods, study designs, and reported performances with related clinical applications on representative studies. The challenges among the reviewed studies were then summarized with discussion.

## 1 INTRODUCTION

Image synthesis across and within medical imaging modalities is an active area of research with broad applications in radiology and radiation oncology. Its primary purpose is to facilitate the clinical workflow by bypassing or replacing an imaging procedure when acquisition is infeasible due to constraints on time, labor, or expense; when exposure to ionizing radiation is contraindicated; or when image registration introduces unacceptable uncertainty between images of different modalities. These benefits have sparked growing interest in a number of exciting clinical applications, such as magnetic resonance imaging (MRI)‐only radiation therapy treatment planning and positron emission tomography (PET)/MRI scanning.

Image synthesis and its potential applications have been investigated for decades. Conventional methods usually rely on models with explicit human‐defined rules for the conversion of images from one modality to another and require case‐by‐case parameter tuning for optimal performance. These models are also application specific, depending upon the unique characteristics of the involved imaging modalities, resulting in a multitude of application‐specific complex methodologies. It is difficult to build such models when the two imaging modalities considered provide distinct information, such as anatomical imaging and functional imaging. This, at least in part, is why the majority of these studies are limited to computed tomography (CT synthesis from MRI).^1^

Owing to the rapid progress in the fields of machine learning and computer vision over the last two decades, image synthesis across other imaging modalities such as PET and cone‐beam CT (CBCT) is now viable and a growing number of applications are benefitting from recent advancements in image synthesis techniques.^2^, ^3^, ^4^ Deep learning, as a broad subdiscipline within machine learning and artificial intelligence, has dominated this field for the past several years. Deep learning utilizes neural networks with many layers containing large numbers of neurons to extract useful features from images. Various networks and architectures have been proposed for better performance on different tasks. Deep learning‐based image synthesis methods usually share a common framework that uses a data‐driven approach for image intensity mapping. The workflow typically consists of a training stage for the network to learn the mapping between the input and its target, and a prediction stage to synthesize the target from an input. Compared with conventional model‐based methods, deep learning‐based methods are more generalizable since the same network and architecture for a pair of image modalities can be generalized to different pairs of image modalities with minimal adjustment. This allows rapid translation to a variety of imaging modalities whose synthesis is clinically useful. Although network training requires significant effort in data collection and curation, prediction usually takes only a few seconds. Due to these advantages, deep learning‐based methods have attracted great research and clinical interest in medical imaging and radiation therapy.

In this paper, we systematically review emerging deep learning‐based methods and applications for medical image synthesis. Specifically, we categorize the recent literature based on their deep learning methods and highlighted their contributions. Clinical applications are surveyed with identification of pertinent limitations and challenges. Finally, recent trends and future directions are summarized.

## 2 LITERATURE SEARCH

We defined the scope of this review study to include both inter‐ and intra‐modality image synthesis using deep learning methods. Inter‐modality applications included studies of image synthesis between two different imaging modalities, whereas intra‐modality applications included studies that transform images between two different protocols of the same imaging modality, such as between MRI sequences, or the restoration of images from a low‐quality protocol to a high‐quality protocol. Studies with a sole aim of image quality improvement, such as image denoising and artifact correction, were not included in this study. Conference abstracts and proceedings were not considered due to the lack of strict peer review in study design and reported results.

**Fig. 1:**
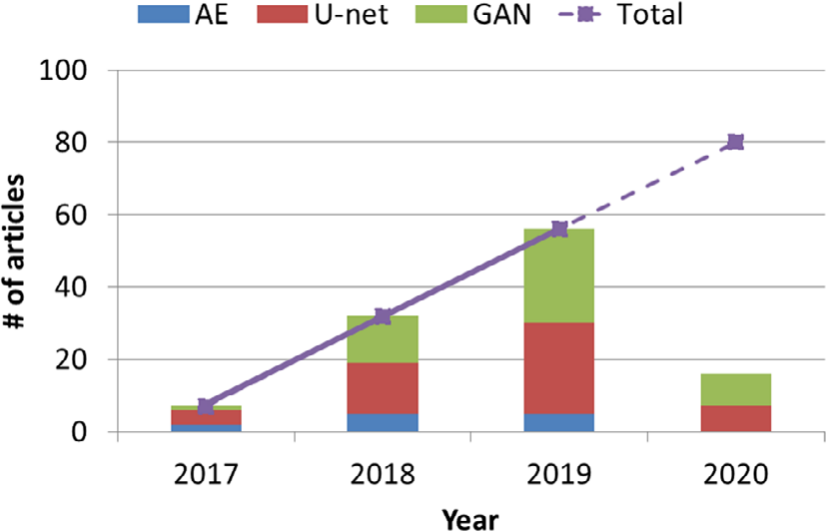
Number of peer‐reviewed articles in medical imaging synthesis using deep learning with different neural networks. This study only covers the first 2 months of 2020. The dashed line predicting the total number of articles in 2020 is a linear extrapolation based on previous years.

Peer‐reviewed journal publications were searched on PubMed using the criteria in title or abstract as of February 2020: (“pseudo” OR “synth*” OR “reconstruct*” OR “transform” OR “restor*” OR “correct*” OR “generat*”) AND “deep” AND “learning” AND (“CT” OR “MR” OR “MRI” OR “PET” OR “SPECT” OR “Ultrasound”). The search yielded 681 records. We manually screened each record, removing those ineligible by the previously defined criteria. The remaining 70 articles were included in this review. We also performed a citation search on the identified literature and an additional 41 articles were included. Therefore, 111 articles were included in this review. Compared with current review papers on this topic,^5^ this review is more comprehensive, covering more articles using a systematic approach. Figure 1 shows the number of reviewed articles by year of publication. The earliest was published in 2017 with an increment of approximately 25 per year, the number of publications on this topic has increased linearly. The number of articles published on this topic in the first 2 months of 2020 has surpassed the total number in 2017.

## 3 DEEP LEARNING METHODS

The methodological frameworks of the reviewed studies can be grouped into three categories: Auto‐encoder (AE), U‐net, and generative adversarial network (GAN). These three groups of methods are not completely different from each other, but represent stepwise increases in architecture complexity. An AE is a basic network and can act as a basic component in advanced architectures such as U‐net and GANs. For example in U‐net, composed of an encoder that downsamples images to feature maps and a decoder that up‐samples the feature maps before finally mapping to targets, the encoder or decoder is usually a fully convolutional AE. Similarly, GANs are commonly viewed as a two‐player zero‐sum game between two neural network architectures: GANs are composed of a generator, which can be an AE or a U‐net, and a discriminator, which is usually an AE. Therefore a hierarchy of complexity can be constructed ranging from the simplest AE to the most complex GAN, with U‐net residing somewhere in between.

**Fig. 2:**
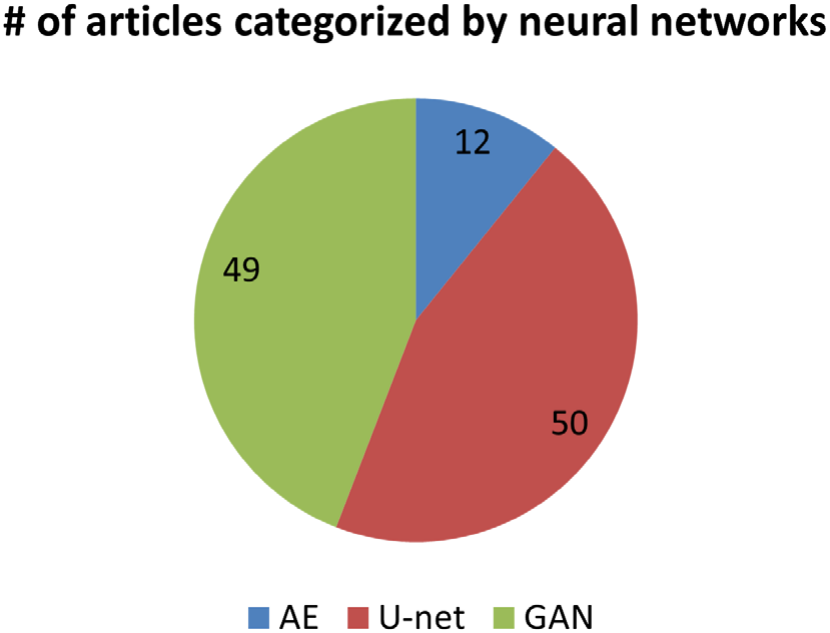
Pie chart of numbers of articles in different categories of neural networks.

Figure 2 indicates that U‐net and GAN studies, which are close in total numbers, comprise the mainstream, accounting for about 90% of the considered articles. Figure 1 also demonstrates that the studies using U‐net and GAN have been increasing since 2017, with GAN utilization increasing at a faster rate than U‐net. While most of the 111 considered studies employ these methods in supervised learning context, three used an unsupervised strategy learning image translation from unpaired datasets. A review of methods within AEs, U‐net, and GANs is provided in this section.

### 3.A Auto‐encoder

An Auto‐encoder (AE) is a class of deep neural networks that use convolution kernels to explore spatially local image patterns. It consists of an input, an output, and multiple hidden layers. The hidden layers contain a series of convolutional layers that convolve the input with trainable convolution kernels and pass the feature maps to the next layer. In order to restrict the input of each convolutional layer into a certain range, an activation layer is added between convolutional layers to map the output of previous layers to the input of the next layers by a predefined function. The Rectified Linear Unit (ReLU) layer which has zero‐valued output for all negative inputs and preserves the input otherwise is the most commonly used activation layer due to its computational simplicity, representational sparsity, and linearity. To further standardize the input of each layer, batch normalization is usually applied to the activations of a layer, rescaling the input to have a standard distribution. This step has been shown to reduce internal covariate shift of the training datasets for improved robustness and faster convergence. Dropout layers are commonly used to reduce the chances of overfitting by intentionally and randomly ignoring some number of layer outputs. In this way, the prior layer is practically implemented with a different number of nodes and connectivity relative to its state before subjecting it dropout. To save memory, the large size of images is typically reduced by pooling and convolution layers to allow a larger number of feature maps and, ultimately, deeper networks. The pooling layer is usually added after the activation layer and involves a pooling operation that uses a specified mathematical filter to downsample the feature map. With multiple hidden convolutional layers, a hierarchy of increasingly complex features with high‐level abstraction is extracted. The ultimate goal is to train the network to minimize the output of an objective loss or cost function: a mathematical representation of the goodness‐of‐fit of the model in matching its predictions to ground truth where greater “loss” or “cost” is associated with poorer fit. During the training process, iterative adjustments are made on the weights and biases of the kernels of the convolutional layers until the loss function is minimized. These weights and biases are trainable parameters of the network. Gradient descent, wherein the mathematical gradient (multi‐dimensional derivative) of the cost function is utilized to minimize the function in a stepwise fashion and update trainable parameters of networks. Several optimization algorithms, such as stochastic gradient descent (SGD), the adaptive gradient algorithm (AdaGrad), root mean square propagation (RMSProp), and Adaptive Moment Estimation (Adam) have been developed. A basic AE is composed of several connected convolutional layers to map input to output; however, very few studies employ AEs in this basic form. Instead, most of the reviewed studies use variants of the basic AE architecture for better performance. For example, the residual neural network (ResNet) was chosen in a few studies due to its shortcut connections that skip one or more layers, easing the training of the deep network without adding extra parameters or computational complexity.^6^, ^7^, ^8^ ResNet also allows feature maps from the initial layers that usually contain fine details to be easily propagated to the deeper layers. AEs and their variants are commonly utilized as a basic component in advanced architectures such as those that follow.

### 3.B U‐net

In one of the first of several studies employing deep learning in image synthesis, Han used AE in synthesizing CT from MR images by adopting and modifying a U‐net architecture.^9^ The U‐net model used in the study of Han has an encoding and a decoding part. In this case, an encoder extracts hierarchical features from an MR image input using convolutional, batch normalization, ReLU, and pooling layers while a mirrored decoder replaces pooling layers with deconvolution layers, transforms the features, and reconstructs the predicted CT images from low‐ to high‐resolution levels. The two parts are connected through shortcuts on multiple layers. These shortcut connections are used to concatenate early layers to late layers such that late layers can also learn simple features captured in early layers. In the study of Han, these shortcuts enable high‐resolution features from the encoding part to be used as extra inputs in the decoding part. Moreover, the original AE design includes several fully connected “hidden layers,” so‐called because these fully connected layers connect every neuron in the previous layer to every neuron in the next and neither inputs nor outputs of these layers are typically monitored during production. The fully connected layers correspond to global image features that are critical for image classification tasks but not very relevant for dense pixel‐wise prediction.^9^ Han’s model removed fully connected layers such that the number of parameters was highly reduced. In their study, the model was trained using pairs of MR and CT two‐dimensional (2D) slices. During the training process, a loss function of mean absolute error (MAE) between prediction and ground truth was minimized. Use of an L1‐norm loss function such as MAE can improve robustness to noise, artifacts, and misalignment among the training images.

Most studies employing U‐net generally followed the above architecture, with many variants and improvements proposed and studied. For example, compared with the model of Han, Jang et al. and Liu et al. applied a similar encoder and decoder model without the skip connection.^10^, ^11^ Instead of using CT images as ground truth in their MR‐based CT study, they used discretized maps from CTs by labeling three materials, transforming CT synthesis into a segmentation problem. Finally, a multi‐class soft‐max classifier giving the probabilities of each material class within each voxel (e.g., 0.5 bone, 0.3 air, 0.1 soft tissue) was applied to the final layer of the decoder. Another notable feature presented in Jang et al.^11^ is inclusion of a fully connected conditional random field, which considers neighboring voxels when generating label predictions, providing complementary information in addition to the base classifier, which only considers single voxel each time. In this application, the conditional random field provided 3D context to 2D image slices, building pairwise potentials between all pairs of voxels using the output of the model and the original 3D volume when predicting the label for each voxel. A landmark advance in U‐net architecture came when Dong et al. discovered that the information carried in the long skip connection of U‐net from the encoding path is characterized by its high frequency, often including irrelevant components from noisy input images. In order to address this issue, they used a self‐attention strategy that uses the feature maps extracted from coarse‐scale early in the encoder module to identify the most relevant emerging features, assign them attention scores and use these to eliminate noise prior to concatenation.^12^ In an alternative strategy, Hwang et al. only employed the skip connection in deeper layers.^13^

The choice of building blocks within the encoding and decoding modules has also been investigated. Fu et al. made a few improvements based on the architecture of Han. For example, batch normalization layers, wherein normalization is applied across image subsets of the original sample to speed convergence, were replaced with instance normalization layers, wherein normalization occurs instead at the level of image channels, for further performance improvements when training with a small batch size. The unpooling layers in the decoder, which up‐sample and therefore reverse pooling layers in the encoder and produce sparse feature maps, were also replaced with deconvolutional layers that produce dense feature maps and the skip connections were replaced with residual shortcuts, inspired by ResNet, to further save computational memory.^14^ Neppl et al. also replaced the ReLU layer with a generalized parametric ReLU (PReLU) to adaptively adjust the activation function.^15^ Torrado‐Carvajal et al. added a dropout layer before the first transposed convolution in the decoder to avoid overfitting.^16^

Various loss functions have been investigated in the reviewed studies. In addition to the most commonly used L1‐norm and L2‐norms that enforce voxel‐wise similarity, other functions that describe different image properties are usually combined into the total loss function. For example, Leynes et al. used a total loss function which was a sum of MAE loss, gradient difference loss, and Laplacian difference loss, the last two of which help improve image sharpness.^17^ Similarly, Chen et al. combines the MAE loss with structure dissimilarity loss to encourage whole‐structure‐wise similarity.^18^ L2‐regularization has also been incorporated into the loss function in a few studies to avoid overfitting.^19^, ^20^ Kazemifar et al. used mutual information, which has been widely implemented in loss functions applied to the task of image registration, in their loss function and demonstrated its advantages over MAE loss in better compensating the misalignment between CT and MR images. Largent et al. introduced a perceptual loss, which can mimic human visual perception using similar features rather than only intensities, into their U‐net. The perceptual loss was proposed in three different implementations with increasing complexity: on a single convolutional layer, on multiple layers with uniform weights, and on multiple layers with different weights that give more importance to the layers yielding the lower MAE.^21^

### 3.C Generative adversarial networks

A generative adversarial network (GAN) is composed of a generative network and a discriminative network that are trained simultaneously. The generative network is trained to generate synthetic images, and the discriminative network is trained to classify an input image as real or synthetic. The training goal of a GAN then is to let the generative network produce synthetic images that are as realistic as possible to fool the discriminator while the discriminative network attempts to distinguish the synthetic from real images. Network training occurs when the adversarial generative and discriminative networks compete against each other until equilibrium is reached. When deployed in production, the trained generative network is applied on new incoming image.

Similar to AEs, GANs were also used in one of the earliest publications in medical image synthesis using deep learning. Nie et al. used a fully convolutional AE (AE without fully connected layers) for the generative network and a standard AE for the discriminative, respectively.^22^ A binary cross‐entropy loss function was employed for both networks with an important distinction: the discriminative network’s loss is formulated to minimize the difference between assigned labels and ground truth in the usual fashion while the generative network’s loss is instead formulated to maximize the error of the discriminative network by minimizing the difference between the labels assigned by the discriminative network and an incorrect label. Since the network in this study was trained in a patch‐to‐patch manner that may limit the context information available in the training samples, an auto‐context model that integrates low‐level and context information from low‐level appearance features was employed to refine the results.

Many variants of the GAN have been designed and investigated. Emami *et al*. adopted conditional GAN (cGAN) in CT synthesis from MR.^7^ Unlike standard unconditional GAN, both the generative and discriminative networks of cGAN observe the input images (e.g., the MR images in CT synthesis from MR). It can be formulated by conditioning the loss function of the discriminator on the input images and has been proved to be more suitable for image‐to‐image translation tasks.^23^ Liang et al. implemented CycleGAN in their CBCT‐based synthetic CT study.^24^ The CycleGAN includes two generators: a CBCT/CT generator and a CT/CBCT generator, as well as two discriminators: a real CT/synthetic CT discriminator and a real CBCT/synthetic CBCT discriminator. In the first cycle, the input CBCT is fed into the CBCT/CT generator to synthesize a CT, then the synthetic CT is fed into the CT/CBCT generator to regenerate a cycle CBCT, which is ideally identical to the input CBCT. The cycle CBCT is compared to the original input CBCT to generate CBCT cycle consistency loss. Meanwhile, the real CT‐synthetic CT discriminator distinguishes between the real CT and the synthetic CT to generate CT adversarial loss, similar to a standard GAN. To encourage one‐to‐one mapping between CT and CBCT, a second cycle transformation from CT to CBCT is performed. The second cycle is same as the first, except the roles of CBCT and CT are swapped, that is, the real CT is fed into the same CT‐CBCT generator to synthesize CBCT, and then the synthetic CBCT is fed into the same CBCT‐CT generator to generate cycle CT. The cycle CT is compared to the real CT to generate CT cycle consistency loss. The real CBCT–synthetic CBCT discriminator distinguishes between the CBCT and the synthetic CBCT to generate CBCT adversarial loss, similar to a standard GAN. Unlike GAN, the CycleGAN couples an inverse mapping network by introducing a cycle consistency loss which enhances the network performance, especially when paired CT/CBCT training image sets are absent. As a result, CycleGAN can tolerate a certain level of misalignment in the paired training dataset. This property of CycleGAN is attractive to inter‐modality synthesis because misalignment in the training datasets is often inevitable due to the difficulty of obtaining exact matching image pairs. In many studies, training images are still paired by registration to preserve quantitative pixel values and reduce baseline geometric mismatch, allowing the network to focus on mapping details and accelerate training.^25^

Varying structures of feature extraction blocks have proven useful for different applications. A group of studies showed that AEs with residual blocks can achieve promising results in image‐transforming tasks where source and target images are largely similar, such as between CT and CBCT, non‐attenuation‐corrected (NAC) PET and attenuation‐corrected (AC) PET, and low‐counting PET and full‐counting PET. Since these pairs of images are similar in appearance but are quantitatively different, residual blocks, composed of a residual connection in combination with multiple hidden layers, were integrated into the network to learn the differences between the pairs. An input bypasses these hidden layers via the residual connection, thus the hidden layers enforces minimization of a residual image between the source and ground truth target images, thereby minimizing noise and artifacts.^25^, ^26^, ^27^, ^28^, ^29^ In contrast, dense blocks concatenate outputs from previous layers rather than using feed‐forward summation as in a standard AE block, capturing multi‐frequency (high and low frequency) information to better represent the mapping from the source image modality to the target image modality. Dense blocks are therefore commonly used in inter‐modality image synthesis such as MR‐to‐CT and PET‐to‐CT.^12^, ^30^, ^31^, ^32^, ^33^, ^34^

Within GANs, the AEs and its variants are commonly used for both generative and discriminative networks. Emami et al. used ResNet for its generative network.^7^ They removed the fully connected layers and added two transposed convolutional layers after residue blocks as deconvolution. Kim et al. combined the U‐net architecture and the residual training scheme in their generative network.^35^ Olberg et al. proposed a deep spatial pyramid convolutional framework that includes an atrous spatial pyramid pooling (ASPP) module in a U‐net architecture. The module performs atrous convolution at multiple rates in parallel such that multi‐scale features can be exploited to characterize a single pixel.^36^ The encoder is then able to capture rich multi‐scale contextual information, which aids image translation. Compared to the generator, the discriminator is typically implemented in a simpler form. A common example consists of a few downsampling convolutional layers followed by a sigmoid activation layer to binarize the output, as proposed by Liu et al.^33^

Generative adversarial networks and its variants incorporate adversarial loss functions in addition to the image quality and accuracy loss functions contained within U‐net. The adversarial term, unlike the reconstruction term that represents image intensity accuracy, reflects the correct or incorrect decision that the discriminator makes on real or synthetic images. In addition to the binary cross‐entropy loss mentioned above or a similar sigmoid cross‐entropy loss, the negative log‐likelihood functions outlined in the original computer vision publication describing GANs are also widely used. However, the training process may suffer from divergence caused by vanishing gradients and mode collapse when the discriminator is trained to be optimal for a fixed generator.^37^ To address this problem, Emami et al. proposed to use least‐square loss, which has been shown to be more stable during training and generates higher quality results.^7^ The Wasserstein distance loss function is an alternative with even smoother gradient flow and faster convergence.^37^ It has also been shown that, in GANs, simply providing the true or false labels output by the discriminator may not be sufficient for the generator to improve, but instead may result in numerical instability during training secondary to vanishing or exploding gradients. Ouyang et al. employed a feature‐matching technique by specifying a new objective function such that the generator encourages the synthesized images to match the expected value of features on the intermediate layers of the discriminator instead of directly maximizing the final output of the discriminator.^38^

### 3.D Other

In addition to the above architectures, other designs have also been proposed to adapt to specific applications in the reviewed studies. For example, Zhang et al. proposed a dual‐domain AE framework that uses two parallel AEs operating in the spatial and frequency domains which interact with each other by Fourier transform to generate synthetic 7T MRI from 3T MRI.^39^ The additional integration of the frequency domain proved to be superior to using the spatial domain alone in synthetic accuracy. In the study of ultralow‐dose amyloid PET reconstruction by Ouyang et al., a pretrained classifier that predicts amyloid status (positive or negative) is incorporated into a GAN‐based network. The pretrained amyloid status classifier acts as a feature extractor, providing feature maps in the calculation of perceptual loss in the GAN.

Using images from multiple modalities as inputs to deep learning networks has been shown to be effective in providing more useful features for learning and testing in several studies. These multi‐modality images are usually treated as inputs with multiple channels in the first layer, each of which has a spatial invariant kernel applied for convolution on the entire image. Wang et al. claimed that the contributions of different modalities could vary at different locations, thus they added a locality adaptive fusion network that takes two modalities (a low‐counting PET and a T1‐weighted MRI) as input to generate a fused image by learning different convolutional kernels at different image locations. The fused image is then fed into the generative network in a GAN architecture.^40^ In contrast to common multichannel inputs in a single path, Tie et al. used three MR images with varying contrast as multichannel inputs in a multipath architecture which has three training paths in the encoder, with each channel possessing its own feature network.^8^ The separate image feature extractions on different MR images are able to avoid the loss of unique features that may be merged in a lower level.

## 4 APPLICATION AREAS

The reviewed articles were categorized into two groups based on their objectives: inter‐modality (56%) and intra‐modality (44%) synthesis. Within each group, subgroups are described that specify the involved imaging modalities and their clinical applications.

### 4.A Inter‐modality

**Fig. 3:**
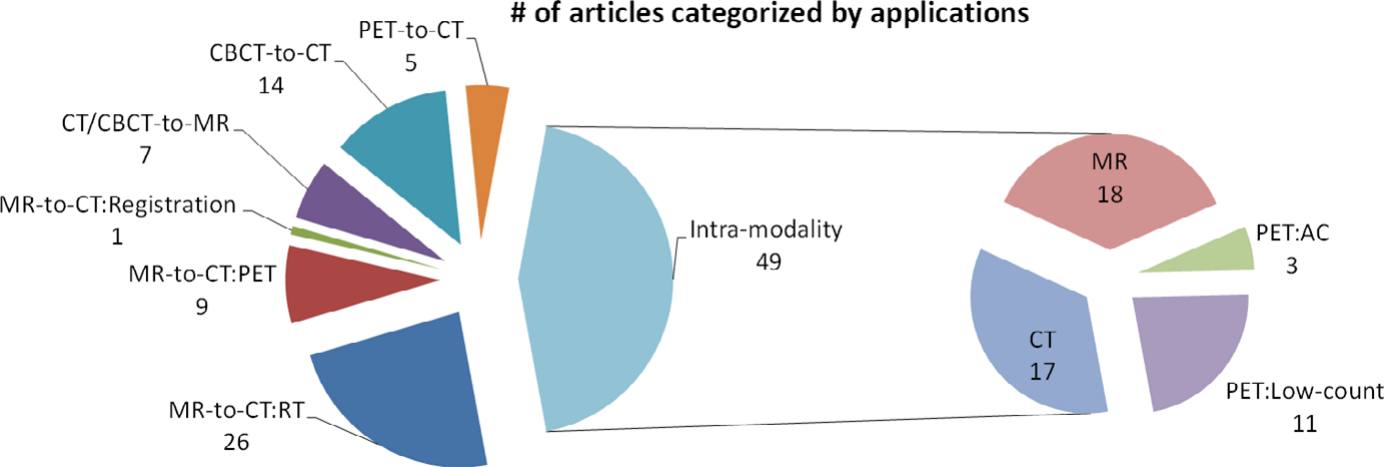
Pie chart of numbers of articles in different categories of applications. MR‐to‐CT: RT, MR‐to‐CT: PET, and MR‐to‐CT: Registration represent MR to CT image synthesis used in radiotherapy, PET, and image registration, respectively. PET: AC and PET: Low‐count represent PET image synthesis used in attenuation correction and low‐count to full‐count, respectively.

The group of inter‐modality synthetic techniques includes studies of image synthesis from one image modality to another, such as from MR to CT, from CT to MR, from PET to CT, etc. We also consider the transformation between CT and CBCT to be inter‐modality because they are acquired from different machines with different hardware and are reconstructed with different principles and algorithms. Studies in this group were further divided into four subgroups: (a) MR‐to‐CT, (b) CT/CBCT‐to‐MR, (c) CBCT‐to‐CT, and (d) PET‐to‐CT. As shown in Fig. 3, MR‐to‐CT synthesis along with its applications in radiation therapy, PET and image registration, accounts for about half of the considered inter‐modality studies and two‐thirds of the studies considered overall.

#### 4.A.1 MR‐to‐CT

Image synthesis from MR to CT is one of the first applications to utilize deep learning for medical image synthesis and remains the most commonly published topic in this field. The main clinical motivation of MR‐based CT synthesis is to replace CT with MR acquisition.^41^ The image quality and appearance of the synthetic CT in current studies is still considerably different from real CT, which prevents its direct diagnostic usage. However, many studies have demonstrated its utility in the nondiagnostic setting, such as treatment planning for radiation therapy and PET attenuation correction.

In the current radiation therapy workflow, both MR and CT imaging are frequently performed on many patients for the purpose of treatment planning (i.e., simulation). MR images feature excellent soft tissue contrast that is useful for delineation of gross tumor as well as organs at risk (OARs)^42^ while CT images provide electron density maps for dose calculation and reference images for pretreatment positioning. The contours from MR images are propagated to CT images by image registration for treatment planning. However, using both imaging modalities not only leads to additional time and cost for the patient, but also introduces systematic positioning errors during the CT‐MRI image fusion process.^43^, ^44^, ^45^ Moreover, CT also subjects patients to exposure to a non‐negligible dose of ionizing radiation,^46^ especially in those requiring resimulation. Thus, it is highly desirable to bypass CT scans in favor of an MRI‐based treatment planning workflow. Emerging MR‐linear accelerator (MR‐linac) technology also motivates the exclusive use of MRI in radiotherapy.^47^, ^48^ Without accurate image synthesis, MR imaging cannot replace CT imaging in radiotherapy because the signal recorded in MR images derives from the hydrogen nucleus and thus cannot provide material attenuation coefficients for electron density calibration and subsequent dose calculation.

Replacing CT with MRI is also preferable in current PET imaging applications, although CT is widely combined with PET in order to perform both imaging examinations simultaneously during a single encounter. The CT images acquired are then used to derive the 511 keV linear attenuation coefficient map to model photon attenuation by a piecewise linear scaling algorithm.^49^, ^50^ The linear attenuation coefficient map is then used to correct for the loss of annihilation photons by attenuation processes in the object on the PET images to achieve a satisfactory image quality. Magnetic resonance has been proposed to be incorporated with PET as a promising alternative to existing PET/CT systems for its advantages of superior soft tissue contrast and radiation dose sparing; however, a challenge similar to that encountered in applications in radiation therapy remains: MR images cannot be directly used to derive the 511 keV attenuation coefficients used in the attenuation correction process. Therefore, MR‐to‐CT image synthesis could be useful to develop a PET/MR system capable of providing the necessary data for photon attenuation correction.^51^

The absence of a one‐to‐one relationship between MR voxel intensity and CT HU values leads to a large difference in image appearance and contrast, which results in failure of intensity‐based calibration methods. For example, bone is bright and air is dark on CT imaging while both are dark on MRI. Conventional methods proposed in the literature either segment MR images into several classes of materials (e.g., air, soft tissue, bone), then assign corresponding CT HU values,^52^, ^53^, ^54^, ^55^, ^56^, ^57^ or register MR images to an atlas with known CT HU values.^58^, ^59^, ^60^ These methods rely heavily on the performance of segmentation and registration, which introduces significant error due to the ambiguous boundary between, for instance, bone and air and large inter‐patient variation.

Tables 1 and 2 list the studies synthesizing CT from MR images for radiation therapy and PET attenuation correction, respectively. For CT synthesis applications in radiation therapy, the MAE is the most common and well‐defined metric by which nearly every study reported the image quality of its synthetic CT. For synthetic CT in PET attenuation correction, synthetic CT quality is more commonly evaluated indirectly by assessing the quality of PET attenuation correction than by direct evaluation of the synthetic CT itself. For studies presenting several variants of methods, we listed that with the best MAE for radiation therapy and the best PET quality for PET attenuation correction.

**Table 1:** Summary of studies on MR‐based synthetic CT for radiation therapy.

Network	MR parameters	Site and # of patients in training/testing	Key findings in image quality	Key findings in dosimetry	Author, year
U‐net	1.5T T1w without contrast	Brain: 18, sixfold cross validation	MAE (HU): 84.8 ± 17.3	N/A^a^	Han, 2017^9^
GAN	N/A	Brain: 16 Pelvis: 22	MAE (HU): 92.5 ± 13.9	N/A	Nie et al., 2018^22^
AE	T1w	Brain: 16, leave‐one‐out Pelvis: 22, leave‐one‐out	MAE (HU): 85.4 ± 9.24 (brain) 42.4 ± 5.1 (Pelvis)	N/A	Xiang et al., 2018^143^
AE	1.5T T1w	Brain: 52, twofold cross validation	MAE (HU): 67 ± 11	Dose difference < 1%	Dinkla et al., 2018^64^
U‐net	3T T2w	Pelvis: 39, fourfold cross validation	MAE (HU): 32.7 ± 7.9	Dose difference < 1%	Arabi et al., 2018^65^
U‐net	3T T2w	Pelvis: 36 training/15 testing	MAE (HU): 29.96 ± 4.87	Dose difference of max dose in PTV < 1.01%	Chen et al., 2018^62^
GAN	1T post‐Gadolinium T1w	Brain: 15, fivefold cross validation	MAE (HU): 89.3 ± 10.3	N/A	Emami et al., 2018^7^
GAN	Dixon in‐phase, fat and water	Pelvis: 91 (59 prostate + 18 rectal + 14 cervical cancer), 32 (prostate) training/59 (rest) testing	MAE (HU): 65 ± 10 (Prostate) 56 ± 5 (Rectum) 59 ± 6 (Cervix)	Dose difference < 1.6%	Maspero et al., 2018^144^
U‐net	3T in‐phase Dixon T2w	Head and neck: 22 training/12 testing	MAE (HU): 75 ± 9	Mead dose difference −0.03%±0.05% overall, ‐0.07%±0.22% in > 90% of prescription dose volume	Dinkla et al., 2019^61^
U‐net	1.5T T1w without contrast	Pelvis: 20, fivefold cross validation	MAE (HU): 40.5 ± 5.4 (2D) 37.6 ± 5.1 (3D)	N/A	Fu et al., 2019^14^
U‐net	3T in‐phase Dixon T1w	Brain: 47 training/13 testing	MAE (HU): 17.6 ± 3.4	Mean target dose difference 2.3 ± 0.1%	Gupta et al., 201 ^63^
GAN	1.5T post‐Gadolinium T1w	Brain: 77, 70% training/12% validation/18% testing	MAE (HU): 47.2 ± 11.0	Mean DVH metrics difference < 1%	Kazemifar et al., 2019^73^
GAN	3T T2w	Pelvis: 39, training/testing: 25/14, 25/14, 25/11	MAE (HU): 34.1 ± 7.5	PTV V95% difference < 0.6%	Largent et al., 2019^21^
CycleGAN	Brain: T1w Pelvis: T2w	Brain: 24, leave‐one‐out cross validation Pelvis: 20, leave‐one‐out cross validation	MAE (HU): 55.7 ± 9.4 (Brain) 50.8 ± 15.5 (Pelvis)	N/A	Lei et al., 2019^31^
U‐net	1.5T T1w	Brain: 30 training/10 testing	MAE (HU): 75 ± 23	PTV V95% difference 0.27% ± 0.79%	Liu et al., 2019^145^
CycleGAN	3T/1.5T T1w	Liver: 21, leave‐one‐out cross validation	MAE (HU): 72.87 ± 18.16	Mean DVH metrics difference < 1% for both photon and proton plans	Liu et al., 2019^32^ and ^34^
CycleGAN	1.5T T2w	Pelvis: 17, leave‐one‐out cross validation	MAE (HU): 51.32 ± 16.91	Mean DVH metrics difference < 1% (Proton plan)	Liu et al., 2019^34^
U‐net	1.5T T1w	Brain: 57 training/28 validation/4 testing	MAE (HU): (82, 147)^b^	Gamma passing rate: >95% at (1%, 1 mm) for photon plan, >90% at (2%, 2 mm) for proton plan	Neppl et al., 2019^15^
GAN	0.35T T1w	Breast: 48 training/12 testing	MAE (HU): 16.1 ± 3.5	PTV D95 difference < 1%	Olberg et al., 2019^36^
CycleGAN	1.5T T1w	Brain: 50	MAE (HU): 54.55 ± 6.81	PTV D95 difference < 0.5% (proton plan)	Shafai‐Erfani et al., 2019^75^
U‐net	1.5T T2w	Head and neck: 23 training/10 testing	MAE (HU): 131 ± 24	N/A	Wang et al., 2019^146^
U‐net	3T T1w Dixon	Pelvis: 27, threefold cross validation	MAE (HU): (33, 40)	N/A	Florkow et al., 2020^71^
GAN	T1w + T2w+ FLAIR	Brain: 15	MAE (HU): 108.1 ± 24.0	DVH metrics difference < 1%	Koike et al., 2020^147^
GAN	T1w + T2w+ Contrast‐enhanced T1w + Contrast‐enhanced T1w Dixon water	Head and neck: 30 training/15 testing	MAE (HU): 69.98 ± 12.02	Mean average dose difference < 1%	Qi et al., 2020^70^
GAN	1.5T Pre contrast T1w + post contrast T1w + T2w	Head and neck: 32, eightfold cross validation	MAE (HU): 75.7 ± 14.6	N/A	Tie et al., 2020^8^
GAN	1.5T and 3T T2w from three scanners	Pelvis: 11 training from two scanner/8 testing from one scanner	MAE (HU): 48.5 ± 6	Maximum dose difference in target = 1.3%	Brou Boni et al., 2020^142^

^a^ N/A: not available, that is, not explicitly indicated in the publication.

^b^ Numbers in parentheses indicate minimum and maximum values.

**Table 2:** Summary of studies on MR‐based synthetic CT for PET attenuation correction.

Network	MR parameters	Site and # of patients in training/testing	Key findings in synthetic CT quality	Key findings in PET quality	Author, year
U‐net	Dixon and ZTE	Brain: 14, leave‐two‐out	MAE (%): 12.62 ± 1.46	Absolute bias < 3% among 8 VOIs	Gong et al., 2018^72^
U‐net (Encoder‐decoder)	3T UTE	Brain: 30 pretraining/6 training/8 testing	N/A^a^	Bias (%): −0.8 ± 0.8 to 1.1 ± 1.3 among 23 VOIs	Jang et al., 2018^11^
U‐net	3T Dixon and ZTE	Pelvis:26, 10 training/16 testing	Mean error (HU): −12 ± 78	RMSE (%): 2.68 among 30 bone lesions, 4.07 among 60 soft‐tissue lesions	Leynes et al., 2018^17^
U‐net (Encoder‐decoder)	1.5T T1w	Brain: 30 training/10 testing	N/A	Bias (%): −3.2 ± 1.3 to 0.4 ± 0.8	Liu et al., 2018^10^
U‐net	1.5T T1w	Brain: 44 training/11 validation/11 testing	Global Bias (%): −1.06 ± 0.81	Global Bias(%): −0.49 ± 1.7 for 11C‐WAY‐100635 −1.52 ± 0.73 for 11C‐DASB	Spuhler et al., 2019^20^
U‐net	Dixon‐VIBE	Pelvis: 28 pairs from 19 patients, fourfold cross validation	MAE (%): 2.36 ± 3.15	Bias (%): 0.27 ± 2.59 for fat −0.03 ± 2.98 for soft tissue −0.95 ± 5.09 for bone	Torrado‐Carvajal et al., 2019^16^
U‐net	ZTE	Brain: 23 training/47 testing	N/A	Bias (%): −1.8 ± 1.9 to 1.7 ± 2.6 among 70 VOIs	Blanc‐Durand et al., 2019^68^
U‐net	UTE	Brain: 79 (pediatric), fourfold cross validation	N/A	Bias (%): −0.2 to 0.5 in 95% CI	Ladefoged et al., 2018^69^
GAN	3T T1w	Brain: 40, twofold cross validation	MAE (HU): 302 ± 79 (bone)	Absolute bias < 4% among 63 VOIs	Arabi et al., 2019^79^

^a^ N/A: not available, that is, not explicitly indicated in the publication.

##### Synthetic CT image accuracy

In most of the studies, the MAE of the synthetic CT within the patient's body ranges from 40 to 70 HU, with some of the reported results approaching uncertainties observed in standard CT simulation. For example, the MAE of soft tissue reported in several studies^7^, ^14^, ^21^, ^61^, ^62^, ^63^, ^64^ is <40 HU. In contrast, due to their indistinguishable contrast on MR images, the MAE of bone or air is more than 100 HU. Another common source of error is misalignment between CT and MR images in the patient datasets. The misalignment that happens on the bone not only causes intensity mapping error during training, but also leads to overestimation of error in evaluation since the error from misalignment registers as synthetic error. Two studies also reported much higher MAE for rectum (~70 HU) than other soft tissue,^21^, ^65^ which may also be attributed to mismatch on CT and MRI due to variable filling. Moreover, considering that the number of bone pixels is far fewer than those of soft tissue, the training process tends to map pixels to low HU region in the prediction stage. Potential solutions may include assigning higher loss weights on bone or adding bone‐only images for training.^14^

Compared with conventional methods, learning‐based methods demonstrate superior performance in synthetic CT accuracy in multiple studies, indicating an advantage of the data‐driven approach over model‐based methods.^9^, ^22^, ^62^, ^65^ For example, synthetic CT generated by atlas‐based methods was shown to be more noisy and prone to registration error, leading to significantly greater MAE than learning‐based methods. However, atlas‐based methods were shown to be more robust to image quality variation in some cases.^65^ One of the limitations of learning‐based methods is that performance can be unpredictable when applied to datasets that are very different from the training sets. These differences may be attributed to unusual or abnormal anatomy or images with degraded quality due to severe artifacts and noise. Atlas‐based methods, in contrast, generate a weighted average of templates from prior knowledge, and are thus less likely to fail on unexpected or unusual cases.

The results reported among these studies cannot be compared directly to determine a single best methodology for all applications because they utilize diverse datasets as well as training and testing strategies. However, some studies compared proposed methods with competing methods using the same datasets, which may reveal their relative advantages and limitations. For example, a GAN‐based method was shown to better preserve detail, be more similar to real CT with less noise compared to a AE‐based method on a cohort of 15 brain cancer patients.^7^ Specifically, GAN‐based synthetic CT was more accurate at the bone/air interface and in determining fine structures, with around 10 HU less error by MAE. Largent et al. compared U‐net and GAN with different loss functions on 39 patients with prostate cancer: U‐net with L2‐norm loss, U‐net with single‐scale perceptual loss, GAN with L2 loss, GAN with single‐scale perceptual loss, GAN with multi‐scale perceptual loss, and GAN with weighted multi‐scale perceptual loss.^21^ Quantitative results showed that the U‐net methods had significantly higher MAE than their GAN counterparts. The perceptual loss in U‐net and GAN did not help decrease MAE, nor provide any benefits for dose calculation accuracy. Lei et al. compared CycleGAN and GAN‐based methods on patients with brain and prostate cancer.^31^ Significant improvement in MAE was observed in the CycleGAN results, with better visual results on fine structural detail and contrast. CycleGAN results, which were less sensitive to local mismatch in the training CT/MR pairs, have less blurry bone boundaries than GAN results. Similar comparison results were also reported by Liu et al., where CycleGAN and GAN were compared on liver stereotactic body radiation therapy (SBRT) cases.^32^ However, dosimetry comparison showed minimal difference, attributed to volumetric‐modulated arc therapy (VMAT) plans which are insensitive to HU inaccuracy.

Among the reviewed studies, several different MR sequences have been adopted for synthetic CT generation. The specific sequence used in each study usually depends upon availability. The optimal sequence yielding the best performance has not, to our knowledge, been studied. T1‐weighted and T2‐weighted sequences are the two most common general diagnostic MR sequences. Due to their wide availability, models can be trained from a relatively large number of datasets with CT and accompanying co‐registered T1‐ or T2‐weighted MR images. T2 images may be preferable to T1 due to their intrinsically superior geometric accuracy within regions of great anatomic variability, such as the nasal cavity, and have less chemical shift artifacts at fat and tissue boundaries. However, air and bone have little contrast in either T1‐ or T2‐weighted MR images, which may impede the extraction of their corresponding features in learning‐based methods. The two‐point Dixon sequence can separate water and fat, which is suitable for segmentation and has already been applied in commercial PET/MR applications with combination of volume‐interpolated breath‐hold examination (VIBE) for Dixon‐based soft tissue and air segmentation for PET attenuation correction as a clinical standard.^66^, ^67^ Its drawback, too, is again the poor contrast of bone, which results in the misclassification of bone as fat. In order to enhance bone contrast and facilitate feature extraction in learning‐based methods, ultrashort echo time (UTE)–and/or zero echo time (ZTE) MR sequences have been recently used to generate positive image contrast from bone.^10^ Ladefoged et al. and Blanc‐Durand et al. demonstrated the feasibility of UTE and ZTE MR sequences using U‐net in PET/MR attenuation correction, repectively.^68^, ^69^ However, neither compared UTE/ZTE to conventional MR sequences under the same deep learning network. Thus, the advantage of this specialized sequence has not been validated. Moreover, compared with conventional T1‐/T2‐weighted MR images, the UTE/ZTE MR images have little diagnostic value on soft tissue and have a long acquisition time, which may reduce their clinical utility in poorly tolerated long‐duration examinations such as whole‐body PET/MR.

Other studies attempted to use multiple MR images with varying contrast as training input in order to provide additional features to the network, intended to enhance overall predictive power and resulting synthetic CT accuracy. Qi et al. proposed a four‐channel input composed of T1, T2, contrast‐enhanced T1, and contrast‐enhanced T1 Dixon water images. Compared with the results from fewer channels, the four‐channel results demonstrated lower MAE.^70^ Florkow et al. investigated single and multichannel input using magnitude MR images and Dixon‐reconstructed water, fat, in‐phase and opposed‐phase images obtained from a single T1 multi‐echo gradient‐echo acquisition.^71^ They found multichannel input is able to improve synthetic CT generation over single‐channel input. Among the multichannel input configurations tested, the Dixon sequence input outperformed all others. Tie et al. used T2 and pre‐ and postcontrast T1 MR images in a multichannel, multipath architecture, demonstrating an additional improvement over multichannel single‐path and single‐channel results.^8^ An attractive combination is UTE/ZTE and Dixon, which provide contrast of bone against air and fat against soft tissue, respectively.^17^, ^72^ Leynes et al. showed that the synthetic CT using ZTE and Dixon MR has less error than that using Dixon alone.^17^ Although the resulting image quality improvement has been validated, the necessity of performing additional MR sequences for the purpose of synthetic CT generation requires further study in specific applications to justify the additional associated costs and acquisition time.

In the reviewed studies, CT and MR images in the training datasets were acquired separately on different machines. Thus, image registration is required between the CT and MR images to create CT‐MR pairs for training. The registration error is minimal at the level of the brain, but may be significant within the pelvis, due to variable bladder and rectum filling, and the abdomen, due to the variation introduced by respiratory motion and peristalsis. U‐net and GAN‐based methods are susceptible to registration error if using a pixel‐to‐pixel loss, which is exacerbated by these types of physiologic motion. Kazemifar et al. proposed a possible solution that uses mutual information as the loss function in the GAN generator to bypass the registration step during training.^73^ As CycleGAN was originally developed for unpaired image‐to‐image translation, CycleGAN‐based methods feature greater robustness to registration error due to the role of the cycle consistency loss in enforcing structural consistency between the original and cycle‐generated images (e.g., enforcing a cycle MRI generated from synthetic CT to be similar to the original MRI).^12^, ^25^, ^27^, ^74^

##### MR‐only radiation therapy

For studies with applications in radiation therapy, many evaluated the dosimetric accuracy of synthetic CT by calculating radiation treatment dose from the original treatment plan and comparing against ground truth CT simulation imaging. It has been shown that the dose difference is approximately 1%, which is small compared to typical total dose delivery uncertainties over an entire treatment course (5%). Compared to image accuracy, the improvement in dosimetric accuracy provided by deep learning‐based methods in radiation therapy is relatively small and may lack clinical relevance.^62^, ^65^ A potential reason is that dose calculation on photon plans is quite forgiving to image inaccuracy, especially within homogeneous regions such as brain. For VMAT, the contribution to dosimetric error from random image inaccuracy also tends to cancel out within an arc. However, the small dosimetric improvement observed may be important in stereotactic radiosurgery (SRS) and SBRT, where small volumes are treated to very high dose. In such cases, significant dosimetric error may arise from otherwise negligible errors in CT synthesis in the region surrounding the target volume.^3^

Studies have also evaluated synthetic CT in the context of proton therapy for prostate, liver, and brain cancer.^33^, ^34^, ^75^ Unlike photon beams, which exhibit a broad dose distribution, proton beams deposit dose with very high‐dose gradient (sharp Bragg peak) at the distal end of the beam. By superimposing proton beams from several angles, this dose distribution characteristic can be exploited to provide highly conformal dose to the target. Any HU inaccuracy along the beam path on the planning CT would lead to shift of the highly conformal high‐dose area, which may cause the tumor to be substantially underdosed or the OARs to be overdosed.^76^ As shown in the Liu et al.,^34^ most of the dose difference resulting from the use of synthetic CT arises at the distal end of the proton beam. As reported by Liu et al.,^33^, ^34^ the largest and mean absolute range difference is 0.56 and 0.19 cm among their 21 liver cancer patients, and 0.75 and 0.23 cm among 17 prostate cancer patients, respectively.

In addition to dosimetric accuracy for treatment planning, another important consideration in the evaluation of synthetic CT imaging is geometric fidelity for treatment setup. Unfortunately, studies on synthetic CT positioning accuracy are sparse. Fu et al. conducted patient alignment testing by rigidly aligning synthetic CT and real CT to the CBCT acquired during delivery of the first fraction of a fractionated radiotherapy treatment course.^14^ The translation vector distance and absolute Euler angle difference between the two alignments were found to be less than 0.6 mm and 0.5° on average, respectively. Gupta et al. conducted a similar study and found the translation difference was less than 0.7 mm in one direction.^63^ Aside from alignment with CBCT, the alignment between the digitally reconstructed radiograph (DRR) derived from the synthetic CT and onboard kilovolt (kV) imaging of the patient is also of clinical interest. However, no study on DRR alignment accuracy is found in the literature reviewed. Note that the geometric accuracy of synthetic CT is not only affected by the synthetic methods employed, but also by the geometric distortion on MR images caused by magnetic field inhomogeneity in addition to subject‐induced susceptibility and chemical shift. Methods to mitigate MR distortion are also important in improving synthetic CT accuracy in patient positioning.

##### PET attenuation correction

For the studies of PET attenuation correction, the bias on PET quantification caused by synthetic CT error has been evaluated. Although it is difficult to specify an error tolerance beyond which clinical decision‐making is impacted, the general consensus is that quantitative errors of 10% or less typically do not affect decisions in diagnostic imaging.^77^ Based on the average relative bias represented by these studies, almost all of the proposed methods in the studies met this criterion. However, it should be noted that, due to variation among study objects, the bias in some volumes‐of‐interest (VOIs) may exceed 10% for some patients,^17^, ^68^ suggesting that attention should be given to the standard deviation of the bias as well as its mean when interpreting results, since the proposed methods may have poor local performance that would affect some patients. Alternative results reporting listing or plotting all data points, or at least their range, would ultimately be more informative than giving a mean and standard deviation in demonstrating the performance of the proposed methods.

Since bone has the highest capacity for attenuation due to its high density and atomic number,^78^ its accuracy on synthetic CT plays a vital role in the final accuracy of attenuation‐corrected PET. Compared to applications in radiation therapy, bias and geometric accuracy of bone on synthetic CT is more often evaluated for PET attenuation correction. Several studies have shown that improved bone accuracy in CT synthesis yields more globally accurate PET.^16^, ^68^, ^72^, ^79^ The more accurate synthetic CT images generated by learning‐based methods therefore lead to more accurate PET attenuation correct. Such improvements were found to be significant in the reviewed studies. It has been shown that PET attenuation correction by conventional CT synthesis methods have about 5% bias on average among selected VOIs while for learning‐based methods the bias was reduced to around 2%.^10^, ^11^, ^16^, ^17^, ^72^

##### MR‐CT image registration

In addition to radiation treatment planning and PET attenuation correction, MR‐based CT synthesis has also proven promising in facilitation of inter‐modality image registration. Direct registration between CT and MR images is very challenging due to disparate image contrast and is even less reliable in deformable registration wherein significant geometric distortion is allowed. McKenzie et al. proposed a CycleGAN‐based method to synthetize CT images and used the synthetic CT to replace MRI in MR‐CT registration in the head and neck, reducing an inter‐modality registration problem to an intra‐modality one.^80^ As summarized in Table 3, they found that, using the same deformable registration algorithm, the average landmark error decreased from 9.8 ± 3.1 mm in direct MR‐CT registration to 6.0 ± 2.1 mm using synthetic CT as a bridge. Similar results were also reported in the inverse CT‐MR registration task.

**Table 3:** Summary of studies on MR‐based synthetic CT for registration.

Network	MR parameters	Site and # of patients in training/testing	Key findings in synthetic CT quality	Key findings in registration accuracy	Author, year
CycleGAN	0.35T	Head and neck: 25, fivefold cross validation	N/A^a^	landmark error (mm): 6.0 ± 2.1 (MR‐to‐CT) 6.6 ± 2.0 (CT‐to‐MR)	McKenzie et al., 2019^80^

^a^ N/A: not available, that is, not explicitly indicated in the publication.

#### 4.A.2 CT/CBCT‐to‐MRI

Due to superior soft tissue contrast produced by MRI, it is attractive to generate synthetic MRI from CT or CBCT in applications that are sensitive to soft tissue contrast, such as segmentation.^81^ Synthesizing MR from CT/CBCT may at first seem more challenging than synthesizing CT from MR, in part because MR contains greater contrast and detail that must be recovered but are not shown on CT; however, deep learning methods have proven quite competent in mapping high nonlinearity, making the proposed application possible.

Studies reviewed synthesizing MR from CT/CBCT‐adopted similar networks to those employed in MR‐to‐CT synthesis and are listed in Table 4. In most of these, the generated synthetic MR served as a bridge to be used in indirect applications, so the image intensity accuracy of the synthetic MR was not reported. In studies that did report synthetic MR accuracy, MAE is less meaningful than other image similarity metrics such as peak signal‐to‐noise ratio (PSNR) since the MR image intensity is relative.

**Table 4:** Summary of studies on CT/CBCT‐based synthetic MR.

Network	MR parameters	Site and # of patients in training/testing	Key findings in synthetic MR quality	Application	Author, year
CycleGAN	T2w	Pelvis: 140, fivefold cross validation	N/A^a^	Male pelvis multi‐organ segmentation on CT	Dong et al., 2019^30^
GAN	3T T2w	Lung: 42 MRIs and 377 CTs, unpaired training	Kullback–Leibler divergence in tumor: 0.069	Augment training data for lung tumor segmentation on MR	Jiang et al., 2019^82^
CycleGAN	3T T2w	Spine: 549 training/92 testing	PSNR(dB): 64.553 ± 1.890	Diagnosis	Jin et al., 2019^148^
CycleGAN	3T T2w	Brain: 192 training/10 testing	PSNR(dB): 65.35	Diagnosis	Jin et al., 2019^149^
GAN	1.5T and 3T T2w	Spine: 280 pairs in training/15 testing	PSNR(dB): 64.9 ± 1.86	Diagnosis	Lee et al., 2020^150^
CycleGAN	1.5T T2w	Pelvis: 49, leave‐one‐out	N/A	Prostate segmentation on CT	Lei et al., 2020^83^
CycleGAN	T2w	Pelvis: 100, fivefold cross validation	N/A	Male pelvis multi‐organ segmentation on CBCT	Lei et al., 2020^84^

^a^ N/A: not available, that is, not explicitly indicated in the publication.

Jiang et al. proposed to use synthetic MR to augment the training data for MR tumor segmentation in lung cancer.^82^ Here, only 81 MR image sets were available with tumor contours delineated by experts, a small sample size for training a segmentation model. In order to enlarge the training dataset, they employed a GAN‐based model to generate synthetic MRI from 377 CT image sets with tumor labeled using other groups of unpaired MR image sets. The 377 synthetic MR image sets with tumor labels were then incorporated into segmentation model training. The addition of synthetic MR in the training set improved segmentation performance, increasing the Dice similarity coefficient (DSC) from 0.50 ± 0.26 to 0.75 ± 0.12. They also showed that, among the synthetic MRIs generated by different methods, the ones more nearly resembling real MR offered better segmentation results. Of note, in the training stage of this model, the ground truth contours for the synthetic MRIs were not delineated on MR, but on CT. Thus, in the testing stage, the output contours were also expected to be CT based. Since the delineation of tumor relies heavily on image contrast, the contour for a single object may vary between CT and MRI. In some cases (prostate cancer, etc.), MR‐based contours are considered the gold standard due to MRIs superior soft tissue contrast relative to CT. Therefore, using CT‐based contours as ground truth in training for MR synthesis may not only confuse the network, but also squander the superior soft tissue contrast provided by MRI.

In the studies of Dong et al. and Lei et al., synthetic MRIs were used instead as a bridge to facilitate segmentation on CT/CBCT images.^30^, ^83^, ^84^ The segmentation targets in their study include prostate, which exhibits low contrast in CT/CBCT but is revealed in high contrast in MRI and tends to be over‐contoured with larger variation on CT/CBCT images when compared with MRI alone or in combination with CT.^85^, ^86^ The synthetic MRIs generated by CT were then aimed at providing superior soft tissue contrast for prostate segmentation. In their studies, paired CT and MRI image sets were used and the prostate contours used as ground truth for MR synthesis were delineated on MR alone or in combination with CT. Compared to ground truth MR‐guided physician contours, synthetic MR‐based prostate segmentation yielded a DSC of 0.87 ± 0.04 compared to 0.82 ± 0.09 with CT alone, a statistically significant improvement.

#### 4.A.3 CBCT‐to‐CT

Cone‐beam CT and CT image reconstruction are subject to the common physics principles of x‐ray attenuation and back projection; however, they differ in the details of their implementation of acquisition and reconstruction as well as their clinical utility. Therefore, they are considered as two distinct imaging modalities in this review.

cOne‐beam CT has been widely utilized in image‐guided radiation therapy (IGRT) to determine the degree of patient setup error and inter‐fraction motion by comparing the displacement of anatomic landmarks from the treatment planning CT images.^87^ With increasing adoption of adaptive radiation therapy techniques, more demanding applications of CBCT have been proposed, such as daily dose estimation and auto‐contouring based on a deformable image registration (DIR) with CT imaging obtained at simulation.^88^, ^89^

Unlike CT scanners using fan‐shaped x‐ray beams with multi‐slice detectors, CBCT generates a cone‐shaped x‐ray beam incident on a flat panel detector. The flat panel detector features a high spatial resolution and wide coverage along the z‐axis, but also suffers from increased scatter signal since the x‐ray scatter generated from the entire body volume may reach the detector. The scatter signals cause severe streaking and cupping artifacts on the CBCT images and lead to significant quantitative CT errors. Such errors complicate the calibration process of CBCT Hounsfield Unit (HU) to electron density when images are used for dose calculation.^90^ The degraded image contrast and suppression of bone can also cause large errors in DIR for contour propagation from planning CT to CBCT.^91^ The significantly degraded image quality of CBCT prevents its use in advanced quantitative applications in radiation therapy.

Deep learning‐based methods, as listed in Table 5, have been proposed to correct and restore CBCT HU values relative to CT by exploiting advantages provided by image translation. CBCT images are reconstructed from hundreds of 2D projections from different angles. A few studies applied a neural network in the projection domain, that is, the 2D projection images, in order to enhance the quality of the projection images prior to volume reconstruction. The quality‐improved projection images were then used to reconstruct CBCT image volume. Others processed in the image domain, that is, directly input the reconstructed CBCT image volumes and output synthetic CT with improved image quality. Projection domain methods can be advantageous in a larger number of training 2D projection images (>300) than training image slices of image domain methods (<100) for each scan. Moreover, the cupping and streaking artifacts caused by scatter on CBCT images are less predictable than those on projection images so that projection images are easier for neural networks to learn. Per‐patient artifactual variation is likewise greater in the image domain, so much so that image domain methods do not typically train models on non‐anthropomorphic phantoms because the data gathered would be useless across patient image sets. However, such variation in image features is not present in the projection domain. As a result, Nomura et al. were able to demonstrate that the features characterizing scatter distribution in anthropomorphic phantom projections can be learned from non‐anthropomorphic phantom projections,^92^ potentially because the neural network successfully learned the inherent relationship between the scatter distribution and objective thickness in the projection domain. The relationship between scatter artifact and objective appearance is much more complex in the image domain and cannot be easily learned.

**Table 5:** Summary of studies on CBCT‐based synthetic CT for radiation therapy.

Network	Projection or image domain	Site and # of patients in training/testing	Key findings in synthetic CBCT quality	Key findings in dosimetry	Author, year
U‐net	Projection	Pelvis: 15 training/7 testing/8 evaluation	MAE (HU): 46	Passing rate for 2% dose difference: 100% for photon plan, 15%–81% for proton plan	Hansen et al., 2018^94^
U‐net	Image	Pelvis: 20, fivefold cross validation	PSNR (dB): 50.9	N/A^a^	Kida et al., 2018^97^
AE	Image	Lung: 15 training/5 testing	PSNR (dB):8.823	N/A	Xie et al., 2018^98^
U‐net	Image	Head and neck: 30 training/7 validation/7 testing Pelvis: 6 training/5 testing	MAE (HU): 18.98 (head and neck) 42.40 (pelvis)	N/A	Chen et al., 2019^18^
CycleGAN	Image	Brain: 24, leave‐one‐out Pelvis: 20, leave‐one‐out	MAE (HU): 13.0 ± 2.2 (brain) 16.1 ± 4.5 (Pelvis)	N/A	Harms et al., 2019^25^
CycleGAN	Image	Pelvis: 18 training/7 validation/8 testing	MAE (HU): 87 (79, 106)^b^	Passing rate for 2% dose difference: 100% for photon plan, 71%‐86% for proton plan	Kurz et al., 2019^93^
U‐net	Image	Pelvis: 27 training/7 validation/8 testing	MAE (HU): 58 (49, 69)	Passing rate for 2% dose difference: >99.5% for photon plan, >80% for proton plan	Landry et al., 2019^95^
U‐net	Image	Head and neck: 50 training/10 validation/10 testing	MAE (HU): (6, 27)	Average DVH metrics difference: 0.2 ± 0.6%	Li et al., 2019^151^
CycleGAN	Image	Head and neck: 81 training/9 validation/20 testing	MAE (HU): 29.85 ± 4.94	Gamma passing rate at (1%, 1mm): 96.26 ± 3.59%	Liang et al., 2019^24^
U‐net	Projection	1800 projections in training (simulation)/200 validation (simulation)/360 testing (phantom)	MAE (HU): 17.9 ± 5.7	N/A	Nomura et al., 2019^92^
U‐net	Image	Head and neck: 33, threefold cross validation	MAE (HU): 36.3 ± 6.2	Gamma passing rate at (2%, 2mm): 93.75‐99.75% (proton)	Adrian et al., 2020^96^
U‐net	Image	Head and neck: 40 training/15 testing	MAE (HU): 49.28	N/A	Yuan et al., 2020^152^
CycleGAN	Image	Pelvis: 16 training/4 testing	Mean error (HU): (2, 14)	N/A	Kida et al., 2020^153^
CycleGAN	Image	Pancreas: 30. Leave‐one‐out	MAE (HU): 56.89 ± 13.84	DVH metrics difference < 1 Gy	Liu et al., 2020^29^

^a^ N/A: not available, that is, not explicitly indicated in the publication.

^b^ Numbers in parentheses indicate minimum and maximum values.

In the reviewed studies, the ground truth considered while training on CBCT images/projections is typically the corresponding CT images/projections captured from the same patient. However, mismatch is commonly seen between CT and CBCT and registration is often required to reduce artifact caused by geometric mismatch. Liu et al. compared the performance of their method when using rigidly and deformably registered CBCT‐CT training data in their pancreas study.^29^ They found that synthetic CT generated from rigidly registered training data had slightly higher MAE (58.45 ± 13.88 HU) than those generated from deformably registered data (56.89 ± 13.84 HU, *P* > 0.05) in addition to less noise and better organ boundaries. Kurz et al. showed that using unmatched CT and CBCT as training data in a CycleGAN without a pixel‐wise loss function is feasible to generate synthetic CT with satisfactory quality.^93^ To bypass the registration step, Hansen et al. and Landry et al. proposed to correct CBCTs by conventional methods first, then use the corrected CBCTs as ground truth. Since the corrected CBCTs maintain the same geometry as the original CBCT, registration is not necessary.^94^, ^95^ However, the synthetic CT quality in this setting is limited by conventional CBCT‐generating methods.

In studies that compared the performance of deep learning methods against conventional CBCT correction methods using the same datasets, learning‐based methods feature better image quality.^25^, ^92^, ^96^, ^97^, ^98^ Adrian et al. found their U‐net‐based method outperformed a deformable registration method and an analytical image‐based method with lower MAE and spatial nonuniformity as well as superior accuracy in bone geometry.^96^ Harms et al. similarly observed less noise and improved subjective similarity of their synthetic CT to real CT when compared to a conventional image‐based correction method.^25^ Conventional correction methods are designed to enhance only a single specific aspect of image quality. By contrast, learning‐based methods are capable of modifying every aspect of image quality to mimic CT, such as noise level, which is not usually considered in conventional methods. A few studies also compared different networks on the same patient datasets, demonstrating that CycleGAN outperforms both GAN and U‐net.^24^, ^29^

Synthetic CTs demonstrate significant improvement over original CBCTs in dosimetric accuracy, and approach planning CT for photon dose calculation. Synthetic CT feasibility in VMAT planning has been evaluated in various body sites by investigating select dose volume histogram (DVH) metrics and dose and/or gamma difference. The figure in Liu et al. demonstrates that large local dosimetric error occurred in regions with severe artifacts. Synthetic CT successfully mitigated these artifacts and the caused dosimetric error.^29^ Compared to photon planning, achieving acceptable dosimetric accuracy with synthetic CT in proton planning is more challenging due to range shift, which may be as great as 5 mm.^93^, ^94^, ^95^

#### 4.A.4 PET‐to‐CT

In a PET‐only scanner where neither CT nor MR is available, attenuation correction is currently conducted by transmission scanning with an external positron source rotated around the patient to measure the attenuation of the body. It is therefore desirable to use non‐attenuation‐corrected (NAC) PET to generate synthetic CT images to provide anatomic information. Moreover, for PET/MR, although MR provides anatomical images, the current atlas or registration‐based methods in MR‐based PET/MR attenuation correction are subject to errors in bone on the derived attenuation map. Deriving the attenuation map from existing NAC PET is therefore an attractive alternative.

Studies in this domain are listed in Table 6. Similar to other studies of synthetic image generation, the synthetic CT images were generated from NAC PET images using a deep learning model trained by pairs of NAC PET and CT images acquired from a PET/CT scanner. Synthesizing CT from NAC PET images is intrinsically challenging since the NAC PET images have much lower spatial resolution than CT and provide little anatomic information. In the studies of Hwang et al., time‐of‐flight information was used to generate a maximum‐likelihood reconstruction using activity and attenuation maps as input since they provide more anatomic information than NAC PET.^13^, ^99^ Despite these challenges, the reported average errors are all within 10% consensus tolerance, competitive with results obtained from MR‐based synthetic CT.

**Table 6:** Summary of studies on PET‐based synthetic CT for PET attenuation correction.

Network	Site and # of patients in training/testing	Key findings in synthetic CT quality	Key findings in PET quality	Author, year
U‐net	Brain: 40, fivefold cross validation	N/A^a^	Average 5% error in activity quantification	Hwang et al., 2018^13^
U‐net	Brain: 100 training/28 testing	MAE (HU): 111 ± 16	Bias: <2% among 28 VOIs	Liu et al., 2018^154^
GAN	Brain: 50 training/40 testing	N/A	Bias: <2.5% among 7 VOIs	Armanious et al., 2019^155^
U‐net	Whole body: 60 training/20 validation/20 testing	Relative error (%): 0.91 ± 3.55 (soft tissue) 0.43 ± 6.80 (bone)	Bias (%): 1.31 ± 3.55 in lesions	Hwang et al., 2019^99^
CycleGAN	Whole body: 80 training/39 testing	MAE (HU): 108.9 ± 19.1	Bias (%): 1.07 ± 9.01 in lesions	Dong et al., 2019^12^

^a^ N/A: not available, that is, not explicitly indicated in the publication.

Whole‐body PET is a critical tool in detecting distant metastases in many malignancies. Most of the reviewed studies of image synthesis in PET developed their proposed methods for brain applications. Although machine learning‐based methods are data driven and not site specific, they may not be readily applicable to the whole body due to the anatomic heterogeneity, activity variance across different organs, and inter‐subject variability. Hwang et al. and Dong et al. investigated learning‐based whole‐body PET attenuation correction using synthetic CT.^12^, ^99^ Both reported average bias within target lesions to be around 1%, which is promising for clinical use. Dong et al. reported average bias within 5% in all selected organs except lungs (>10%) in both studies. Poor performance for lung was attributed to tissue inhomogeneity and insufficient representative training data. They also found that synthetic CT demonstrated blurriness in lung, like respiratory motion artifacts that were not shown on CT, indicating that synthetic CTs are more matched to PET than CT and may be more suitable for attenuation correction. Both studies utilized a PET‐only scanner and, so far, there are no learning‐based methods developed for the PET/MR whole‐body scanner. Compared with PET alone, the PET/MR provides additional anatomic structural information from MR, but the integration of MR into PET attenuation correction introduces additional challenges in the whole‐body setting relative to brain due to limited field of view (FOV), longer scan time introducing more motion, and degraded image quality due to a larger inhomogeneous‐field region.

### 4.B Intra‐modality

The group of intra‐modality investigations includes studies that transform images between two different protocols within an imaging modality, such as among different sequences of MRIs, or the restoration of images from a low‐quality protocol to higher quality. Studies solely aiming at image quality improvement such as image denoising and artifact correction are not included in this study. Studies within this group are further subdivided into CT, MR, and PET. As shown in Fig. 3, the number of published studies addressing each of the three imaging modalities is similar.

#### 4.B.1 CT

Computed tomography imaging delivers a non‐negligible dose of ionizing radiation during acquisition leading to a small, but real, increase in risk of radiation‐induced cancer and genetic defects.^100^, ^101^, ^102^ During diagnosis, treatment and surveillance of many malignancies, it is common for patients to be subject to frequent CT imaging. In this setting, accumulated imaging dose is of even greater concern, particularly for pediatric patients who are more sensitive to radiation and have longer life expectancy than adults throughout which secondary malignancies are more likely to develop.^103^

Computed tomography dose can be lowered by either reducing x‐ray exposure (mAs)^104^, ^105^, ^106^, ^107^ or the number of x‐ray projections.^104^, ^105^, ^106^, ^107^ However, if reconstructing an image with a conventional filtered‐backprojection (FBP) algorithm, image quality would be degraded with greater image noise and reduced signal‐to‐noise ratio for a low‐exposure protocol, or with severe undersampling artifacts for a reduced projection protocol. These low‐quality images would make routine tasks requiring CT images difficult for clinicians. Hardware‐based methods such as optimization of the data acquisition protocol (automatic exposure control)^108^ and improvements in detector designs^109^ have been shown to be effective in reducing imaging dose to some extent while maintaining clinically acceptable image quality. However, further dose reduction from these techniques is limited by detector physical properties and is therefore very costly.

For decades, iterative CT image reconstruction algorithms have been proposed to address the degraded image quality resulting from insufficient data acquisition.^110^ These methods model the physical process of CT scanning with prior knowledge and are more robust to noise, requiring less radiation dose for the same image quality relative to FBP.^110^, ^111^, ^112^ However, iterative reconstruction suffers from long computation time due to the large number of iterations with repeated forward and back projection steps. Moreover, in the forward projection step, it requires knowledge of the energy spectrum which is difficult to measure directly.^113^, ^114^, ^115^, ^116^ This is usually addressed by a monoenergetic forward projection matrix, or by obtaining an indirect simulation/estimation of the energy spectrum.^106^, ^107^, ^112^, ^117^, ^118^

Image synthesis by deep learning is attractive for low‐dose CT (LDCT) restoration due to its data‐driven approach to automatically learning image features and model parameters. As listed in Table 7, most of the methods in the reviewed literature implement direct image translation from low‐dose to full‐dose CT while others restore the sinogram using deep learning first, and then reconstruct images from the restored sinogram by FBP. As shown by Dong et al., their proposed projection‐based method outperformed an image‐based method by better reducing downsampling artifacts with higher resolution on object edges.^119^ A potential reason for this difference is that for image‐based methods prediction error is directly observed on the image while for projection‐based methods, the error predicted on the sinogram will be compensated for in the reconstruction process, where the final product is a weighted sum of all sinograms. Projection‐based methods are therefore inherently more robust to error. It is also possible to train a model to map directly from the projection domain to the image domain, with the network encoding a mapping between polar and Cartesian coordinates.^120^ Among image domain methods, Shan et al. used a progressive scheme that iteratively denoised the input LDCT, yielding a sequence of denoised images at different noise levels.^121^ Kang et al. mapped their wavelet coefficient instead of directly mapping low‐ and full‐dose CT images. The benefit of wavelet transformation was revealed in better structure recovery than that achieved with direct image mapping.^122^

**Table 7:** Summary of studies on synthetic full dose CT from low‐dose CT.

Network	Projection or image domain	Site and # of patients in training/testing	Low‐dose scheme and fraction of full dose CT	Key findings in restored full dose CT	Author, year
U‐net	Image	Abdomen: 10 training/20 testing	Low mAs: 1/4 of full dose	PSNR (dB): about 36	Kang et al., 2017^122^
U‐net	Image	Abdomen: 10, leave‐one‐out	Low mAs: 1/4 of full dose	PSNR (dB): 44.4187 ± 1.2118	Chen et al., 2017^156^
U‐net	Image	Thorax and pelvis: 475 slices training/25 slices testing	Sparse view: 1/20 of full views	PSNR (dB): 28.83	Jin et al., 2017^120^
GAN	Image	Cardiac: 28, twofold cross validation	Low mAs: 20% dose	Significantly reduced noise	Wolterink et al., 2017^157^
AE (ResNet)	Image	Abdomen: 9 training/1 testing	Low mAs: 1/4 of full dose	PSNR (dB): 39.8329	Yang et al., 2017^158^
AE (ResNet)	Image	Abdomen: 8 training/1 testing	Low mAs: 1/4 of full dose	PSNR (dB): 38.70	Kang et al., 2018^159^
GAN	Image	Abdomen (piglet): 708 slices training/142 slices testing	Low mAs: 5% of full mAs	PSNR (dB): about 34	Yi and Babyn, 2018^124^
GAN	Image	Abdomen: 5 training/5 testing	Low mAs: 1/4 of full dose	PSNR(dB): 30.137 ± 1.938	Shan et al., 2018^160^
GAN	Image	Abdomen: 10, leave‐one‐out	Low mAs: 1/4 of full dose	PSNR (dB): (25.372, 27.398)^a^	You et al., 2018^161^
U‐net	Image	Abdomen: 8 training/1 validation/1 testing	Sparse view: 1/12 of full views	PSNR (dB): 40.4856	Han and Ye, 2018^162^
GAN	Image	Abdomen: 4000 slices training/2000 testing	Low mAs: 1/4 of full dose	Validated in double‐blinded reader study	Yang et al., 2018^37^
U‐net (Encoder‐decoder)	Image	Whole body: 300 slices training/50 slices testing	Low mAs: fraction not specified	PSNR (dB): 42.3257	Liu and Zhang, 2018 ^163^
AE	Image	Chest: 3 training/3 testing	Low mAs: 3% of full mAs	PSNR (dB): about 22	Zhao et al., 2019^164^
U‐net	Projection	Chest: 7 training/8 testing	Sparse view: 1/4 of full views	PSNR (dB): (42.73, 52.14)	Lee et al., 2019^165^
U‐net	Image	Abdomen and chest: 10 training/60 testing	Low mAs: about 1/3 to 1/8 of full dose	Validated in double‐blinded reader study	Shan et al., 2019^121^
U‐net	Projection	Head: 200 slices training/100 slices testing	Sparse view: 1/12 of full views Limited angle: 1/4 of full views	PSNR (dB): 37.21 for sparse view 43.69 for limited angle	Dong et al., 2019^119^
CycleGAN	Image	Head: 30, fivefold cross validation	Low mAs: 0.5% of full mAs	NMSE (%): 1.63 ± 0.62	Wang et al., 2019^28^

^a^ Numbers in parentheses indicate minimum and maximum values.

Compared with iterative reconstruction methods, learning‐based methods require less time and no prior knowledge about the energy spectrum. For example, as reported by Wang et al., it took about 1 min to generate an entire 3D volume from denoised LDCT images on an average personal computer after their model was trained. In contrast, with the same hardware, a compressed sensing‐based iterative method takes 1 min in forward and back projecting on a single slice in one iteration. Alternatively, if the forward and back projecting operation is precalculated and saved as a sparse matrix, the time can be shortened to several seconds for each slice in each iteration, but requires 6.8 GB in memory to store the matrix. Even so, to reconstruct the entire volume requires several hours. Conventional iterative reconstruction methods are therefore very resource intensive, limiting their implementation on personal computers, especially when slice thickness is small and FOV is large.^28^

Conventional iterative reconstruction methods were compared with learning‐based methods in several studies. For example, total variation (TV) regularization is commonly studied in state‐of‐the‐art compressed sensing‐based iterative methods. A common finding is that TV‐based methods tend to over‐smooth and present patchy textures while the results obtained by learning‐based methods have finer structures preserved and more closely resemble a full‐dose CT in image texture.^28^, ^120^ Such improvement with learning‐based methods is also shown in quantitative metrics of PSNR, etc. Superior recovery of image quality with preserved texture could be attributed to the analytic optimization objectives in machine learning that incentivize model predictions to match ground truth examples (here, full‐dose CT images) in image quality and texture. Similarly, Shan et al. demonstrated that their proposed learning‐based method performed favorably or comparably to three commercially available iterative algorithms in terms of noise suppression and structural fidelity by double‐blinded reader study.

Most of the reviewed studies assume diagnostic applications of their restored full‐dose CT images. Wang et al. evaluated their method in the context of radiation therapy treatment planning^28^ because LDCT employed in the CT simulation process is attractive for adaptive radiation therapy, wherein iterative rescanning and replanning throughout the treatment course is common. In contrast to diagnostic CT, which emphasizes high spatial resolution and low‐contrast lesion detectability, planning CT requires accurate HU numbers and dose calculation accuracy. Their dosimetric study showed that the average differences of DVH metrics between the synthetic full‐dose CT and original full‐dose CT are less than 0.1 Gy (*P* > 0.05) when prescribing to a dose of 21 Gy.

Many of the reviewed studies used the dataset from the AAPM 2016 Low‐Dose CT Grand Challenge.^123^ Although the training and testing strategies may be different among these studies, the results are comparable. However, due to the lack of clinical LDCT data, this LDCT dataset, along with the datasets in most other studies, are simulated from full‐dose CT by adding Poisson noise or downsampling the sinogram. Exceptions include Yi et al., who used piglet subjects and Shan et al., who used real patient LDCTs.^121^, ^124^ Simulated noise may not fully reflect the properties of true noise and potential artifacts, thus it is of clinical interest to evaluate these methods against physically measured LDCT datasets.

#### 4.B.2 MRI

Image synthesis has been investigated for various applications in MRI,^125^ including translation between sequence types, converting low‐magnetic‐field MRI to high‐magnetic‐field MRI, and restoring undersampled acquisitions. Converting low‐magnetic‐field MRI to high‐magnetic‐field MRI allows acquisition on broadly available low‐magnetic‐field equipment while providing greater spatial resolution and improved contrast, similar to what might be obtained from a cutting‐edge scanner while translation between sequences and restoration of undersampled acquisitions can both shorten acquisition times. Although these applications are motivated by distinct clinical goals, they pose the same technical challenges during the task of image synthesis: preserving contrast and resolution.

A large group of conventional methods exist that address these problems. Compressed sensing (CS) methods assume that images have a sparse representation in some transformation domain. For example, in image synthesis between multi‐contrast MR images, an image patch in the source contrast level is expressed as a sparse linear combination of patches in an atlas and the combination is then applied to image patches in the target contrast. In recovering undersampled acquisitions, the problem is usually modeled as a reconstruction problem with regularization terms that incorporate prior knowledge about the sparsity of images. Optimization is usually implemented as an iterative algorithm, which is time and resource intensive. Deep learning methods, in contrast, encourage the integration of neural networks into these strategies for their superior mapping capability of nonlinear relationships and significant savings in compute time.

The related studies are listed in Table 8. Compared with applications in other modalities, more studies of MR inter‐modality synthesis implement neural networks in combination with other techniques, rather than adopting an end‐to‐end deep learning strategy. It is also common to apply neural networks in the transformation domains. Zhang et al. proposed a cascaded regression using two parallel and interactive multi‐layer network streams in the spatial and frequency domains. Compared with a single spatial domain, the dual domain method presented better visual results and a significantly greater structural similarity index measure (SSIM).^39^ Qu et al. designed a wavelet‐based affine transformation layer to modulate feature maps from the spatial and wavelet domains in the encoder, followed by an image reconstruction in the decoder that synthesizes 7T images from wavelet‐modulated spatial information. Without such a layer, the framework was reduced to a simple encoder‐decoder network, which was found to be less capable in recovering detail.^126^

**Table 8:** Summary of studies on synthetic MRI.

Network	Applications	Site and # of patients in training/testing	Key findings in results	Author, year
GAN	Synthesizing 7T MRI from 3T MRI	Brain: 15, leave‐one‐out	PSNR (dB): 27.6 ± 1.3	Nie et al., 2018^22^
AE	Restoring undersampled acquisition	Cardiac: 5 training/5 testing	Restored images showed most of the anatomical structures up to 11‐fold undersampling	Schlemper et al., 2018^127^
GAN	Low resolution to high resolution	Brain: 196 training/48 testing	SSIM: (0.76, 0.94)^a^ at eightfold undersampling	Kim et al., 2018^35^
U‐net	Synthesizing full contrast‐enhanced images from low contrast‐enhanced images	Brain: 10 training/ 50 testing	PSNR (dB): 28.07 ± 2.26 at tenfold lower	Gong et al., 2018^166^
U‐net	T1w to T2w T1w to FLAIR PDw to T2w	Three different brain datasets: 22 training/3 validation/3 testing 42 training/6 validation/6 testing 22 training/3 validation/3 testing	Average PSNR (dB) among groups of datasets: (25.78, 32.92) for synthetic T2w (29.99, 30.32) for synthetic FLAIR	Chartsias et al., 2018^167^
GAN	Restoring undersampled acquisition	Brain and chest: for each site, 100 slices training/100 slices testing	PSNR (dB) at 10% undersampling: about 32 for brain, 26.5 for chest	Quan et al., 2018^168^
GAN	Low resolution to high resolution	767 training/192 validation/30 testing	Average PSNR (dB): about (25, 30)	Galbusera et al., 2018^169^
	T1w to T2w	767 training/192 validation/30 testing		
	T2w to T1w	767 training/192 validation/30 testing		
	T2w to STIR	284 training/71 validation/30 testing		
	T2w to TIRM	305 training/77 validation/30 testing		
GAN	T1w to T2w T2w to T1w	Brain: 3 datasets, 48 training/5 validation/13 testing, 25 training/5 validation/10 testing, 24 training/2 validation/15 testing	Average PSNR (dB): (25.80 ± 1.87, 29.77 ± 1.57) among three datasets	Dar et al., 2019^170^
GAN	Restoring undersampled acquisition	Abdomen: 336 training/10 testing	SSIM: 0.84 at fivefold undersampling	Mardani et al., 2019^171^
U‐net	Synthesizing DTI from fMRI	Brain: 648 training/293 testing	Mean correlation coefficient: 0.808 ± 0.054 among 38 VOIs	Son et al., 2019^19^
AE	Synthesizing FLAIR from mpMRI	Brain: 24, fivefold cross validation	SSIM: 0.860 ± 0.031	Wei et al., 2019^172^
GAN	Synthesizing diffusion b0 maps from T1w	Brain: 586 training/26 testing	Distortion correction based on synthesized b0 maps is feasible	Schilling et al., 2019^133^
AE (ResNet)	Synthesizing arterial spin labeling images from T1w	Brain: 355, fivefold cross validation	Accuracy in CBF calculation and dementia disease diagnosis is close to gold standard	Huang et al., 2019^173^
AE	Synthesizing 7T MRI from 3T MRI	Brain: 15, leave‐one‐out	SSIM: 0.8438	Zhang et al., 2019^39^
U‐net	Restoring undersampled acquisition	Knee: 90 training/10 validation/10 testing	SSIM: 0.821 ± 0.023 at eightfold undersampling	Liu et al., 2019^174^
U‐net (encoder‐decoder)	Synthesizing 7T MRI from 3T MRI	Brain: 15, leave‐one‐out	PSNR (dB): 28.27	Qu et al., 2020^126^
U‐net	Restoring undersampled acquisition	Knee: 336 training/24 testing	SSIM: 0.8603 at fourfold undersampling	Wu et al., 2020^175^
U‐net	Synthesizing MR angiography from 3D‐QALAS sequence	Brain: 11, fivefold cross validation	PSNR (dB): 35.3 ± 0.5	Fujita et al., 2020^176^

^a^ Numbers in parentheses indicate minimum and maximum values.

Many of the reviewed studies also compared their proposed strategies with CS‐based methods with comparable or better performance on quantitative image quality metrics and much less compute time. Predictions carried out in production for deep learning models are typically calculated on the order of milliseconds to seconds while CS‐based methods process in minutes. Schlemper et al. found that at low undersampling rate, learning‐based and CS‐based methods had comparable performance while the advantages of learning‐based methods become evident at more aggressive undersampling factors.^127^ Other suboptimal findings, including loss of detail and blocky artifacts were also reported.

#### 4.B.3 PET

Image synthesis among different PET images has been proposed to facilitate PET attenuation correction and low‐count PET reconstruction. For PET attenuation correction, unlike those summarized in Section 4.A.4 where synthetic CT is generated from NAC PET for attenuation correction during PET reconstruction, a few studies listed in Table 9 investigated the feasibility of directly mapping NAC PET to attenuation‐corrected PET by exploiting deep learning methods to bypass PET reconstruction. These studies reported comparable results with synthetic CT‐based PET attenuation correction (Section 4.A.4), although direct comparisons on single datasets were not found. Dong et al. is credited with direct NAC PET‐AC PET mapping across the whole body for the first time.^26^ They also demonstrate the reliability of their method by including sequential scans in their testing datasets to evaluate the PET intensity changes with time on their attenuation‐corrected PET as well as on ground truth images. Similar to their study using synthetic CT, the greatest error was observed in lung. Shiri *et al*. further assessed radiomic features on their attenuation‐corrected PET results, and found only three of 83 regions demonstrated significant differences from ground truth images.^128^

**Table 9:** Summary of studies on synthetic AC PET from NAC PET.

Network	Site and # of patients in training/testing	Key findings in PET quality	Author, year
U‐net	Brain: 25 training/10 testing	Bias (%): 4.0 ± 15.4	Yang et al., 2019^177^
U‐net	Brain: 91 training/18 testing	Bias (%): −0.10 ± 2.14 among 83 VOIs	Shiri et al., 2019^128^
CycleGAN	Whole body: 25 training/ 10 patients*3 sequential scan testing	Bias (%): (−17.02,3.02)^a^ among 6 VOIs, 2.85 ± 5.21 in lesions	Dong et al., 2019^26^

^a^ Numbers in parentheses indicate minimum and maximum values.

Low‐count PET has extensive applications in pediatric PET scanning and radiotherapy response evaluation with the advantages of better motion control and lower radiation dose. However, low‐count statistics result in increased image noise, reduced contrast‐to‐noise ratio, and significant bias in uptake measurement. The reconstruction of a standard‐ or full‐count PET from low‐count PET cannot be achieved by simple postprocessing operations such as denoising, since the diminished radiation dose changes the underlying biological and metabolic processes, leading not only to noise but also local uptake values changes.^129^ Moreover, even given the same radiotracer injection dose, the uptake distribution and signal level can vary greatly among patients. The learning‐based low‐count PET reconstruction methods summarized in Table 10 have been proposed to take advantage of the powerful data‐driven feature extraction capabilities of neural networks applied across two image datasets. A few of the reviewed methods used both MR and low‐count PET as input while most used low‐count PET alone. Most were implemented on PET of the brain, with a few on lung and whole body. Compared with evaluations of PET attenuation correction which focus on relative bias, the evaluations in the reviewed studies of low‐count PET reconstruction exhibit a greater focus on image quality and the similarity between the predicted result and its corresponding full‐count ground truth counterpart.

**Table 10:** Summary of studies on synthetic full‐count PET from low‐count PET.

Network	PET or PET + MR	Image or projection domain	Site and # of patients in training/testing	Counting fraction (low/full)	Key findings in restored full‐counting PET	Author, year
AE	PET + MR	Image	Brain: 16, leave‐one‐out	1/4	PSNR (dB): 24.76	Xiang et al., 2017^132^
GAN	PET	Image	Brain: 16, leave‐one‐out	1/4	PSNR (dB): about 24	Wang et al., 2018^178^
U‐net	PET + MR	Image	Brain: 40, fivefold cross‐validation	1/100	PSNR (dB): about 38	Chen et al., 2019^131^
AE	PET	Image	Brain: 2 training/1 testing Lung: 5 training/1 testing	Brain: 1/5 Lung: 1/10	N/A	Gong et al., 2019^179^
U‐net (encoder‐decoder)	PET	Projection	Whole body (simulation): 245 training/52 validation/53 testing	N/A	PSNR (dB): 34.69	Haggstrom et al., 2019^180^
GAN	PET + MR	Image	Brain: 16, leave‐one‐out	¼	PSNR (dB): 24.61	Wang et al., 2019^181^
GAN	PET	Image	Brain: 40, fourfold cross validation	1/100	PSNR (dB): about 30	Ouyang et al., 2019^38^
U‐net	PET	Image	Lung: 10, fivefold cross validation	1/10	Bias: <15%	Lu et al., 2019^182^
GAN	PET	Image	Whole body: 435 slices training/440 slices testing	1/10	PSNR (dB): 30.557	Kaplan and Zhu, 2019^183^
CycleGAN	PET	Image	Whole body: 25 training/10 testing	1/8	PSNR (dB): 41.5 ± 2.5	Lei et al., 2019^27^
U‐net	PET	Projection	Brain: 100 training/20 validation/20 testing	1/20	PSNR (dB): 38.25 ± 0.66	Sanaat et al., 2020^130^

Similar to LDCT restoration, most low‐count PET restoration studies apply neural networks directly to the image domain, with a few operating in the projection domain. In addition to the advantages mentioned in Section 4.B.1, Sanaat et al. commented that projection‐based networks allow modification of the reconstruction filter or postprocessing without retraining the model.^130^ They also compared results using original images and projections as input and found that projection‐based results better reflect uptake pattern and anatomy than image‐based results, with both subjective and objective studies validating the advantages of projection‐based results. A drawback of projection‐based methods, however, is training time that is sixfold greater than that required for image‐based methods.

Although these studies demonstrate the feasibility of mapping low‐count PET to full‐count PET, a few investigated using PET and MRI in combination as dual input channels to further improve results when MR images are available. As expected, the additional anatomic information provided by MRI improved the performance of the network observed when trained on PET alone. Chen et al. showed that their network is able to achieve 83% accuracy when using PET as sole input vs 89% when using PET in combination with MR in a clinical reading study of uptake status.^131^ A potential reason for this improvement derives from the superior reflection of anatomic patterns provided by PET and MR together. The contribution of MR images was also validated in the study of Xiang et al. showing a significant improved PSNR.^132^ They commented that structural information from MRIs yields important cues for estimating the high‐quality PET, even though structural MRI differs from PET significantly in overall appearance.

## 5 SUMMARY AND OUTLOOK

Recent years have witnessed the trend in deep learning being increasingly used in the application of medical imaging. The latest networks and techniques have been borrowed from the field of computer vision and adapted to specific clinical tasks in radiology and radiation oncology. As reviewed in this paper, learning‐based image synthesis is an emerging and active field — all the reviewed studies were published within the last 3 yr. With further development in both artificial intelligence and computing hardware, more learning‐based methods are expected to facilitate the clinical workflow with novel applications. Although the reviewed literature show the success of deep learning‐based image synthesis in various applications, there remain some open questions to be answered in future studies.

Due to limitations of GPU memory, some of the deep learning approaches examined were trained on two‐dimensional (2D) slices. Since the loss functions of 2D models do not account for continuity in the third dimension, slice discontinuities can be observed. Some studies trained models on three‐dimensional (3D) patches to exploit 3D spatial information with even less memory burden,^31^ while a potential drawback is that the larger scale image features may be hard to extract.^61^ Training on 3D image stacks is expected to achieve a more homogeneous conversion result. Fu et al. compared the performance of 2D and 3D models using the same U‐net implementation.^14^ They found 3D‐generated synthetic CT exhibited smaller MAE and more accurate bone regions. However, to achieve robust performance, 3D model needs more training data to learn more parameters. A compromise is to use multiple adjacent slices that allow the model to capture more 3D context, or to train different networks for all three combinations of orthogonal 2D planes to produce pseudo‐3D information.^133^

The reviewed studies illustrate the advantages of learning‐based methods over conventional methods in performance as well as clinical application. Learning‐based methods generally outperform conventional methods in generating more realistic synthetic images with higher similarity to real images and better quantitative metrics. Depending on hardware, training a model in development usually takes hours to days for learning‐based methods. However, once the model is trained, it can be applied to new patients to generate synthetic images in seconds to minutes. Conventional methods vary widely in specific methodologies and implementations, resulting in a wide range of run times. Iterative methods such as CS were shown to be unfavorable due to significant costs in time and compute power.

Unlike conventional methods, learning‐based methods require large training datasets. The size of training sets has been shown to affect the performance of machine learning in many challenging computer vision problems as well as medical imaging tasks.^134^, ^135^, ^136^, ^137^ Generally, a larger training set size with greater data variation can reduce overfitting of the model and enable better performance. Compared with studies in some medical imaging applications where it is common to see thousands of patients enrolled, studies in medical image synthesis involve far fewer patients. As shown in Tables 1, 2, 3, 4, 5, 6, 7, 8, 9, 10, a training size of dozens of patients is more common in these studies while hundreds of patients per set are rare and can be considered as a relatively “large” study. Moreover, it is very common to see the leave‐X‐out or N‐fold cross validation strategy used in evaluating methods. The lack of an independent test set unseen by the model may complicate the generalization of results for broad clinical applications. The current small sample norm arises from circumstances which vary from application to application. In radiation oncology, clinical patient volume is inherently lower than other specialties such as radiology, so that fewer eligible patients are available for study. In addition to limitations in data collection, data cleaning further eliminates a portion of data that are low in quality or represent outliers, such as image pairs with suboptimal registration. In order to address the problem posed by limited training data, novel techniques have been proposed, such as transfer learning,^138^ self‐supervised/weakly supervised/unsupervised learning,^139^, ^140^ and data augmentation.^141^ These methods either diminish or completely eliminate dependence on training data sample size; although they may not be applicable to all medical image synthesis applications.

In the training stage, most of the reviewed studies require paired datasets, that is, the source image and target image must exhibit pixel‐to‐pixel correspondence. This requirement poses difficulties in collecting sufficient eligible datasets and demands high accuracy in image registration. Some networks such as CycleGAN can relax the requirement for paired image datasets, which can be beneficial to clinical applications enrolling large number of patients for training.

Although the advantages of learning‐based methods have been demonstrated, it should be noted that their performance can be unpredictable when input images during production differ significantly from training images. In most of the reviewed studies, unusual cases are excluded. However, unusual cases may be realistically observed in the clinical setting and, in these cases, the application of learning‐based methods should be approached with diligence and caution. For example, hip prostheses create severe artifacts on both CT and MR images, thus, it is of clinical and practical interest to understand the effect of their inclusion in training or testing datasets for learning‐based models, but this effect has not yet been studied. Similar unusual cases may also be encountered in other forms in other imaging modalities and are worthy of investigation, such as medical implants that introduce artifacts, obesity resulting in greater image noise, and anatomic deformities or abnormalities.

Due to the limitation in the number of available datasets, most studies used N‐fold cross validation or the leave‐N‐out strategy. The small to intermediate number of patients in training and testing datasets is appropriate for feasibility studies, but is not sufficient for evaluating clinical utility. Moreover, the representativeness of training/testing datasets relative to a particular clinic’s population requires special attention in clinical study. Suboptimal demographic diversity may reduce the robustness and generalizability of any model. Most studies reviewed here trained models using data from a single institution with a single scanner. Model performance across hardware of several models or manufacturers, wherein image characteristics cannot be exactly matched, is an important consideration due to frequent hardware replacement and upgrade in the modern clinical setting. Boni *et al*. recently presented a proof‐of‐concept study that predicted synthetic images of one clinical site using a model trained on data from two other sites and demonstrated clinically acceptable results.^142^ Further studies could include datasets from multiple centers and adopt a leave‐one‐center‐out training and/or test strategy in order to validate the consistency and robustness of the network.

Before being deployed into clinical workflow, there are still several challenges to be addressed. To account for potentially unpredictable synthetic images that can result from noncompliance with imaging protocols in training data or unexpected anatomic variation, additional quality assurance (QA) steps would be essential in clinical practice. QA procedures would aim to check the consistency of model performance routinely or after upgrading by retraining the network with additional patient datasets and verify synthetic image quality on specific cases.

## AUTHORS CONTRIBUTION

**Tonghe Wang** involved in conceptualization, methodology, investigation, writing original draft, and visualization. **Yang Lei and Yabo Fu** involved in conceptualization, methodology, and writing, reviewing, and editing the original draft. **Jacob F. Wynne** involved in writing, reviewing, and editing the original draft. **Walter J. Curran and Tian Liu** involved in writing, reviewing, and editing the original draft and supervision. **Xiaofeng Yang** involved in conceptualization, writing, reviewing, and editing the original draft, supervision, project administration, and funding acquisition.

## CONFLICT OF INTEREST

None.

## References

[1] Johnstone E , Wyatt JJ , Henry AM , et al. Systematic review of synthetic computed tomography generation methodologies for use in magnetic resonance imaging‐only radiation therapy. Int J Radiat Oncol Biol Phys. 2018;100:199–217. 29254773 10.1016/j.ijrobp.2017.08.043

[2] Wang T , Lei Y , Manohar N , et al. Dosimetric study on learning‐based cone‐beam CT correction in adaptive radiation therapy. Med Dosim. 2019;44:e71–e79. 30948341 10.1016/j.meddos.2019.03.001 PMC6773528

[3] Wang T , Manohar N , Lei Y , et al. MRI‐based treatment planning for brain stereotactic radiosurgery: dosimetric validation of a learning‐based pseudo‐CT generation method. Med Dosim. 2019;44:199–204. 30115539 10.1016/j.meddos.2018.06.008 PMC7775641

[4] Yang X , Wang T , Lei Y , et al. MRI‐based attenuation correction for brain PET/MRI based on anatomic signature and machine learning. Phys Med Biol. 2019;64:025001. 30524027 10.1088/1361-6560/aaf5e0 PMC7773209

[5] Yu B , Wang Y , Wang L , Shen D , Zhou L . Medical image synthesis via deep learning. Adv Experim Med Biol. 2020;1213:23–44. 10.1007/978-3-030-33128-3_2 32030661

[6] Missert AD , Yu L , Leng S , Fletcher JG , McCollough CH . Synthesizing images from multiple kernels using a deep convolutional neural network. Med Phys. 2020;47:422–430. 31714999 10.1002/mp.13918

[7] Emami H , Dong M , Nejad‐Davarani SP , Glide‐Hurst CK . Generating synthetic CTs from magnetic resonance images using generative adversarial networks. Med Phys. 2018;45:3627–3636. 10.1002/mp.13047 PMC6294710 29901223

[8] Tie X , Lam SK , Zhang Y , Lee KH , Au KH , Cai J . Pseudo‐CT generation from multi‐parametric MRI using a novel multi‐channel multi‐path conditional generative adversarial network for nasopharyngeal carcinoma patients. Med Phys. 2020;47:1750–1762. 32012292 10.1002/mp.14062

[9] Han X . MR‐based synthetic CT generation using a deep convolutional neural network method. Med Phys. 2017;44:1408–1419. 28192624 10.1002/mp.12155

[10] Liu F , Jang H , Kijowski R , Bradshaw T , McMillan AB . Deep learning MR imaging‐based attenuation correction for PET/MR imaging. Radiology. 2018;286:676–684. 28925823 10.1148/radiol.2017170700 PMC5790303

[11] Jang H , Liu F , Zhao G , Bradshaw T , McMillan AB . Technical note: Deep learning based MRAC using rapid ultrashort echo time imaging. Med Phys. 2018;45:3697–3704. 10.1002/mp.12964 PMC6443501 29763997

[12] Dong X , Wang T , Lei Y , et al. Synthetic CT generation from non‐attenuation corrected PET images for whole‐body PET imaging. Phys Med Biol. 2019;64:215016. 31622962 10.1088/1361-6560/ab4eb7 PMC7759014

[13] Hwang D , Kim KY , Kang SK , et al. Improving the accuracy of simultaneously reconstructed activity and attenuation maps using deep learning. J Nucl Med. 2018;59:1624–1629. 29449446 10.2967/jnumed.117.202317

[14] Fu J , Yang Y , Singhrao K , et al. Deep learning approaches using 2D and 3D convolutional neural networks for generating male pelvic synthetic computed tomography from magnetic resonance imaging. Med Phys. 2019;46:3788–3798. 31220353 10.1002/mp.13672

[15] Neppl S , Landry G , Kurz C , et al. Evaluation of proton and photon dose distributions recalculated on 2D and 3D Unet‐generated pseudoCTs from T1‐weighted MR head scans. Acta Oncol (Stockholm Sweden). 2019;58:1429–1434. 10.1080/0284186X.2019.1630754 31271093

[16] Torrado‐Carvajal A , Vera‐Olmos J , Izquierdo‐Garcia D , et al. Dixon‐VIBE deep learning (DIVIDE) pseudo‐CT synthesis for pelvis PET/MR attenuation correction. J Nucl Med. 2019;60:429–435. 30166357 10.2967/jnumed.118.209288 PMC6910626

[17] Leynes AP , Yang J , Wiesinger F , et al. Zero‐echo‐time and dixon deep pseudo‐CT (ZeDD CT): direct generation of pseudo‐CT images for pelvic PET/MRI attenuation correction using deep convolutional neural networks with multiparametric MRI. J Nucl Med. 2018;59:852–858. 29084824 10.2967/jnumed.117.198051 PMC5932530

[18] Chen L , Liang X , Shen C , Jiang S , Wang J . Synthetic CT generation from CBCT images via deep learning. Med Phys. 2019;47:1115–1125. 10.1002/mp.13978 PMC7067667 31853974

[19] Son SJ , Park BY , Byeon K , Park H . Synthesizing diffusion tensor imaging from functional MRI using fully convolutional networks. Comput Biol Med. 2019;115:103528. 31743880 10.1016/j.compbiomed.2019.103528

[20] Spuhler KD , Gardus J 3rd , Gao Y , DeLorenzo C , Parsey R , Huang C . Synthesis of patient‐specific transmission data for PET attenuation correction for PET/MRI neuroimaging using a convolutional neural network. J Nucl Med. 2019;60:555–560. 30166355 10.2967/jnumed.118.214320

[21] Largent A , Barateau A , Nunes JC et al Comparison of deep learning‐based and patch‐based methods for pseudo‐CT generation in MRI‐based prostate dose planning. Int J Radiat Oncol Biol Phys. 2019;105:1137–1150. 31505245 10.1016/j.ijrobp.2019.08.049

[22] Nie D , Trullo R , Lian J , et al. Medical image synthesis with deep convolutional adversarial networks. IEEE Trans Bio‐Med Eng. 2018;65:2720–2730. 10.1109/TBME.2018.2814538 PMC6398343 29993445

[23] Isola P , Zhu J‐Y , Zhou T , Efros AA .Image‐to‐Image Translation with Conditional Adversarial Networks. arXiv e‐prints. 2016.arXiv:1611.07004. https://ui.adsabs.harvard.edu/abs/2016arXiv161107004I. Accessed November 01, 2016.

[24] Liang X , Chen L , Nguyen D , et al. Generating synthesized computed tomography (CT) from cone‐beam computed tomography (CBCT) using CycleGAN for adaptive radiation therapy. Phys Med Biol. 2019;64:125002. 31108465 10.1088/1361-6560/ab22f9

[25] Harms J , Lei Y , Wang T , et al. Paired cycle‐GAN‐based image correction for quantitative cone‐beam computed tomography. Med Phys. 2019;46:3998–4009. 31206709 10.1002/mp.13656 PMC7771209

[26] Dong X , Lei Y , Wang T , et al. Deep learning‐based attenuation correction in the absence of structural information for whole‐body PET imaging. Phys Med Biol. 2019;65:055011. 10.1088/1361-6560/ab652c PMC7099429 31869826

[27] Lei Y , Dong X , Wang T , et al. Whole‐body PET estimation from low count statistics using cycle‐consistent generative adversarial networks. Phys Med Biol. 2019;64:215017. 31561244 10.1088/1361-6560/ab4891 PMC7764437

[28] Wang T , Lei Y , Tian Z , et al. Deep learning‐based image quality improvement for low‐dose computed tomography simulation in radiation therapy. J Med Imaging. 2019;6:043504. 10.1117/1.JMI.6.4.043504 PMC6811730 31673567

[29] Liu Y , Lei Y , Wang T , et al. CBCT‐based synthetic CT generation using deep‐attention cycleGAN for pancreatic adaptive radiotherapy. Med Phys. 2020;47:2472–2483. 32141618 10.1002/mp.14121 PMC7762616

[30] Dong X , Lei Y , Tian S , et al. Synthetic MRI‐aided multi‐organ segmentation on male pelvic CT using cycle consistent deep attention network. Radiother Oncol. 2019;141:192–199. 31630868 10.1016/j.radonc.2019.09.028 PMC6899191

[31] Lei Y , Harms J , Wang T , et al. MRI‐only based synthetic CT generation using dense cycle consistent generative adversarial networks. Med Phys. 2019;46:3565–3581. 31112304 10.1002/mp.13617 PMC6692192

[32] Liu Y , Lei Y , Wang T , et al. MRI‐based treatment planning for liver stereotactic body radiotherapy: validation of a deep learning‐based synthetic CT generation method. Br J Radiol. 2019;92:20190067. 31192695 10.1259/bjr.20190067 PMC6724629

[33] Liu Y , Lei Y , Wang Y , et al. Evaluation of a deep learning‐based pelvic synthetic CT generation technique for MRI‐based prostate proton treatment planning. Phys Med Biol. 2019;64:205022. 31487698 10.1088/1361-6560/ab41af PMC7765705

[34] Liu Y , Lei Y , Wang Y , et al. MRI‐based treatment planning for proton radiotherapy: dosimetric validation of a deep learning‐based liver synthetic CT generation method. Phys Med Biol. 2019;64:145015. 31146267 10.1088/1361-6560/ab25bc PMC6635951

[35] Kim KH , Do WJ , Park SH . Improving resolution of MR images with an adversarial network incorporating images with different contrast. Med Phys. 2018;45:3120–3131. 29729006 10.1002/mp.12945

[36] Olberg S , Zhang H , Kennedy WR , et al. Synthetic CT reconstruction using a deep spatial pyramid convolutional framework for MR‐only breast radiotherapy. Med Phys. 2019;46:4135–4147. 31309586 10.1002/mp.13716

[37] Yang Q , Yan P , Zhang Y , et al. Low‐dose CT image denoising using a generative adversarial network with wasserstein distance and perceptual loss. IEEE Trans Med Imaging. 2018;37:1348–1357. 29870364 10.1109/TMI.2018.2827462 PMC6021013

[38] Ouyang J , Chen KT , Gong E , Pauly J , Zaharchuk G . Ultra‐low‐dose PET reconstruction using generative adversarial network with feature matching and task‐specific perceptual loss. Med Phys. 2019;46:3555–3564. 31131901 10.1002/mp.13626 PMC6692211

[39] Zhang Y , Yap PT , Qu L , Cheng JZ , Shen D . Dual‐domain convolutional neural networks for improving structural information in 3T MRI. Magn Reson Imaging. 2019;64:90–100. 31175927 10.1016/j.mri.2019.05.023 PMC6874896

[40] Wang Y , Zhou L , Wang L , et al. Locality Adaptive Multi‐modality GANs for High‐Quality PET Image Synthesis. Paper presented at: Med Image Comput Comput Assist Interv; Sep, 2018;11070:329–337. 10.1007/978-3-030-00928-1_38 PMC6494468 31058275

[41] Yang X , Lei Y , Shu H‐K , et al. Pseudo CT estimation from MRI using patch‐based random forest. Paper presented at: Medical Imaging 2017: Image Processing 2017;10133:101332Q. 10.1117/12.2253936 PMC6788808 31607771

[42] Khoo VS , Joon DL . New developments in MRI for target volume delineation in radiotherapy. Br J Radiol. 2006;79:S2–S15. 16980682 10.1259/bjr/41321492

[43] Nyholm T , Nyberg M , Karlsson MG , Karlsson M . Systematisation of spatial uncertainties for comparison between a MR and a CT‐based radiotherapy workflow for prostate treatments. Radiat Oncol. 2009;4:54. 19919713 10.1186/1748-717X-4-54 PMC2781017

[44] Ulin K , Urie MM , Cherlow JM . Results of a multi‐institutional benchmark test for cranial CT/MR image registration. Int J Radiat Oncol Biol Phys. 2010;77:1584–1589. 20381270 10.1016/j.ijrobp.2009.10.017 PMC2906611

[45] van der Heide UA , Houweling AC , Groenendaal G , Beets‐Tan RG , Lambin P . Functional MRI for radiotherapy dose painting. Magn Reson Imaging. 2012;30:1216–1223. 22770686 10.1016/j.mri.2012.04.010 PMC5134673

[46] Devic S . MRI simulation for radiotherapy treatment planning. Med Phys. 2012;39:6701–6711. 23127064 10.1118/1.4758068

[47] Lagendijk JJW , Raaymakers BW , Raaijmakers AJE , et al. MRI/linac integration. Radiother Oncol. 2008;86:25–29. 18023488 10.1016/j.radonc.2007.10.034

[48] Fallone BG , Murray B , Rathee S , et al. First MR images obtained during megavoltage photon irradiation from a prototype integrated linac‐MR system. Med Phys. 2009;36:2084–2088. 19610297 10.1118/1.3125662

[49] Kinahan PE , Townsend DW , Beyer T , Sashin D . Attenuation correction for a combined 3D PET/CT scanner. Med Phys. 1998;25:2046–2053. 9800714 10.1118/1.598392

[50] Burger C , Goerres G , Schoenes S , Buck A , Lonn AH , Von Schulthess GK . PET attenuation coefficients from CT images: experimental evaluation of the transformation of CT into PET 511‐keV attenuation coefficients. Eur J Nucl Med Mol Imaging. 2002;29:922–927. 12111133 10.1007/s00259-002-0796-3

[51] Wang T , Lei Y , Fu Y , et al. Machine learning in quantitative PET: a review of attenuation correction and low‐count image reconstruction methods. Physica Med. 2020;76:294–306. 10.1016/j.ejmp.2020.07.028 PMC7484241 32738777

[52] Lee YK , Bollet M , Charles‐Edwards G , et al. Radiotherapy treatment planning of prostate cancer using magnetic resonance imaging alone. Radiother Oncol. 2003;66:203–216. 12648793 10.1016/s0167-8140(02)00440-1

[53] Jonsson JH , Karlsson MG , Karlsson M , Nyholm T . Treatment planning using MRI data: an analysis of the dose calculation accuracy for different treatment regions. Radiat Oncol. 2010;5:62. 20591179 10.1186/1748-717X-5-62 PMC2909248

[54] Lambert J , Greer PB , Menk F , et al. MRI‐guided prostate radiation therapy planning: investigation of dosimetric accuracy of MRI‐based dose planning. Radiother Oncol. 2011;98:330–334. 21339009 10.1016/j.radonc.2011.01.012

[55] Kristensen BH , Laursen FJ , Løgager V , Geertsen PF , Krarup‐Hansen A . Dosimetric and geometric evaluation of an open low‐field magnetic resonance simulator for radiotherapy treatment planning of brain tumours. Radiother Oncol. 2008;87:100–109. 18262669 10.1016/j.radonc.2008.01.014

[56] Johansson A , Karlsson M , Nyholm T . CT substitute derived from MRI sequences with ultrashort echo time. Med Phys. 2011;38:2708–2714. 21776807 10.1118/1.3578928

[57] Hsu S‐H , Cao Y , Huang K , Feng M , Balter JM . Investigation of a method for generating synthetic CT models from MRI scans of the head and neck for radiation therapy. Phys Med Biol. 2013;58:8419. 24217183 10.1088/0031-9155/58/23/8419 PMC3886820

[58] Dowling JA , Lambert J , Parker J , et al. An atlas‐based electron density mapping method for magnetic resonance imaging (MRI)‐alone treatment planning and adaptive MRI‐based prostate radiation therapy. Int J Radiation Oncol Biol Phys. 2012;83:e5–e11. 10.1016/j.ijrobp.2011.11.056 22330995

[59] Uh J , Merchant TE , Li Y , Li X , Hua C . MRI‐based treatment planning with pseudo CT generated through atlas registration. Med Phys. 2014;41:051711. 24784377 10.1118/1.4873315 PMC5148041

[60] Sjölund J , Forsberg D , Andersson M , Knutsson H . Generating patient specific pseudo‐CT of the head from MR using atlas‐based regression. Phys Med Biol. 2015;60:825. 25565133 10.1088/0031-9155/60/2/825

[61] Dinkla AM , Florkow MC , Maspero M , et al. Dosimetric evaluation of synthetic CT for head and neck radiotherapy generated by a patch‐based three‐dimensional convolutional neural network. Med Phys. 2019;46:4095–4104. 31206701 10.1002/mp.13663

[62] Chen S , Qin A , Zhou D , Yan D . Technical note: U‐net‐generated synthetic CT images for magnetic resonance imaging‐only prostate intensity‐modulated radiation therapy treatment planning. Med Phys. 2018;45:5659–5665. 30341917 10.1002/mp.13247

[63] Gupta D , Kim M , Vineberg KA , Balter JM . Generation of synthetic CT images from MRI for treatment planning and patient positioning using a 3‐channel U‐Net trained on sagittal images. Front Oncol. 2019;9:964. 31608241 10.3389/fonc.2019.00964 PMC6773822

[64] Dinkla AM , Wolterink JM , Maspero M , et al. MR‐only brain radiation therapy: dosimetric evaluation of synthetic CTs generated by a dilated convolutional neural network. Int J Radiat Oncol Biol Phys. 2018;102:801–812. 30108005 10.1016/j.ijrobp.2018.05.058

[65] Arabi H , Dowling JA , Burgos N , et al. Comparative study of algorithms for synthetic CT generation from MRI: consequences for MRI‐guided radiation planning in the pelvic region. Med Phys. 2018;45:5218–5233. 30216462 10.1002/mp.13187

[66] Freitag MT , Fenchel M , Baumer P , et al. Improved clinical workflow for simultaneous whole‐body PET/MRI using high‐resolution CAIPIRINHA‐accelerated MR‐based attenuation correction. Eur Radiol. 2017;96:12–20. 10.1016/j.ejrad.2017.09.007 29103469

[67] Izquierdo‐Garcia D , Hansen AE , Förster S , et al. An SPM8‐based approach for attenuation correction combining segmentation and nonrigid template formation: application to simultaneous PET/MR brain imaging. J Nucl Med. 2014;55:1825–1830. 25278515 10.2967/jnumed.113.136341 PMC4246705

[68] Blanc‐Durand P , Khalife M , Sgard B , et al. Attenuation correction using 3D deep convolutional neural network for brain 18F‐FDG PET/MR: Comparison with Atlas, ZTE and CT based attenuation correction. PLoS One. 2019;14:e0223141. 31589623 10.1371/journal.pone.0223141 PMC6779234

[69] Ladefoged CN , Marner L , Hindsholm A , Law I , Hojgaard L , Andersen FL . Deep learning based attenuation correction of PET/MRI in pediatric brain tumor patients: evaluation in a clinical setting. Front Neurosci. 2018;12:1005. 30666184 10.3389/fnins.2018.01005 PMC6330282

[70] Qi M , Li Y , Wu A , et al. Multi‐sequence MR image‐based synthetic CT generation using a generative adversarial network for head and neck MRI‐only radiotherapy. Med Phys. 2020;47:1880–1894. 32027027 10.1002/mp.14075

[71] Florkow MC , Zijlstra F , Willemsen K et al Deep learning‐based MR‐to‐CT synthesis: the influence of varying gradient echo‐based MR images as input channels. Magn Reson Med. 2020;83:1429–1441. 31593328 10.1002/mrm.28008 PMC6972695

[72] Gong K , Yang J , Kim K , El Fakhri G , Seo Y , Li Q . Attenuation correction for brain PET imaging using deep neural network based on Dixon and ZTE MR images. Phys Med Biol. 2018;63:125011. 29790857 10.1088/1361-6560/aac763 PMC6031313

[73] Kazemifar S , McGuire S , Timmerman R , et al. MRI‐only brain radiotherapy: assessing the dosimetric accuracy of synthetic CT images generated using a deep learning approach. Radiother Oncol. 2019;136:56–63. 31015130 10.1016/j.radonc.2019.03.026

[74] Lei Y , Harms J , Wang T , et al. MRI‐only based synthetic CT generation using dense cycle consistent generative adversarial networks. Med Phys. 2019;46:3565–3581. 31112304 10.1002/mp.13617 PMC6692192

[75] Shafai‐Erfani G , Lei Y , Liu Y , et al. MRI‐based proton treatment planning for base of skull tumors. Int J Particle Ther. 2019;6:12–25. 10.14338/IJPT-19-00062.1 PMC6986397 31998817

[76] Li B , Lee HC , Duan X , et al. Comprehensive analysis of proton range uncertainties related to stopping‐power‐ratio estimation using dual‐energy CT imaging. Phys Med Biol. 2017;62:7056–7074. 28678019 10.1088/1361-6560/aa7dc9 PMC5736379

[77] Hofmann M , Steinke F , Scheel V , et al. MRI‐based attenuation correction for PET/MRI: a novel approach combining pattern recognition and atlas registration. J Nucl Med. 2008;49:1875–1883. 18927326 10.2967/jnumed.107.049353

[78] Yang X , Fei B . Multiscale segmentation of the skull in MR images for MRI‐based attenuation correction of combined MR/PET. J Am Med Inform Assoc. 2013;20:1037–1045. 23761683 10.1136/amiajnl-2012-001544 PMC3822115

[79] Arabi H , Zeng G , Zheng G , Zaidi H . Novel adversarial semantic structure deep learning for MRI‐guided attenuation correction in brain PET/MRI. Eur J Nucl Med Mol Imaging. 2019;46:2746–2759. 31264170 10.1007/s00259-019-04380-x

[80] McKenzie EM , Santhanam A , Ruan D , O'Connor D , Cao M , Sheng K . Multimodality image registration in the head‐and‐neck using a deep learning‐derived synthetic CT as a bridge. Med Phys. 2019;47:1094–1104. 10.1002/mp.13976 PMC7067662 31853975

[81] Yang X , Wu N , Cheng G , et al. Automated segmentation of the parotid gland based on atlas registration and machine learning: a longitudinal MRI study in head‐and‐neck radiation therapy. Int J Radiat Oncol Biol Phys. 2014;90:1225–1233. 25442347 10.1016/j.ijrobp.2014.08.350 PMC4362545

[82] Jiang J , Hu YC , Tyagi N , et al. Cross‐modality (CT‐MRI) prior augmented deep learning for robust lung tumor segmentation from small MR datasets. Med Phys. 2019;46:4392–4404. 31274206 10.1002/mp.13695 PMC6800584

[83] Lei Y , Dong X , Tian Z , et al. CT prostate segmentation based on synthetic MRI‐aided deep attention fully convolution network. Med Phys. 2020;47:530–540. 31745995 10.1002/mp.13933 PMC7764436

[84] Lei Y , Wang T , Tian S , et al. Male pelvic multi‐organ segmentation aided by CBCT‐based synthetic MRI. Phys Med Biol. 2020;65:035013. 31851956 10.1088/1361-6560/ab63bb PMC7042793

[85] Villeirs GM , Van Vaerenbergh K , Vakaet L , et al. Interobserver delineation variation using CT versus combined CT + MRI in intensity‐modulated radiotherapy for prostate cancer. Strahlentherapie und Onkologie. 2005;181:424–430. 15995835 10.1007/s00066-005-1383-x

[86] Pathmanathan AU , McNair HA , Schmidt MA , et al. Comparison of prostate delineation on multimodality imaging for MR‐guided radiotherapy. Br J Radiol. 2019;92:20180948. 30676772 10.1259/bjr.20180948 PMC6540870

[87] Barney BM , Lee RJ , Handrahan D , Welsh KT , Cook JT , Sause WT . Image‐guided radiotherapy (IGRT) for prostate cancer comparing kV imaging of fiducial markers with cone beam computed tomography (CBCT). Int J Radiat Oncol Biol Phys. 2011;80:301–305. 20864274 10.1016/j.ijrobp.2010.06.007

[88] Zhu L , Xie Y , Wang J , Xing L . Scatter correction for cone‐beam CT in radiation therapy. Med Phys. 2009;36:2258–2268. 19610315 10.1118/1.3130047 PMC2832067

[89] de la Adam Z , Benjamin A , Lei X . Formulating adaptive radiation therapy (ART) treatment planning into a closed‐loop control framework. Phys Med Biol. 2007;52:4137. 17664599 10.1088/0031-9155/52/14/008

[90] Marchant TE , Moore CJ , Rowbottom CG , MacKay RI , Williams PC . Shading correction algorithm for improvement of cone‐beam CT images in radiotherapy. Phys Med Biol. 2008;53:5719. 18824785 10.1088/0031-9155/53/20/010

[91] Hou J , Guerrero M , Chen W , D'Souza WD . Deformable planning CT to cone‐beam CT image registration in head‐and‐neck cancer. Med Phys. 2011;38:2088–2094. 21626941 10.1118/1.3554647

[92] Nomura Y , Xu Q , Shirato H , Shimizu S , Xing L . Projection‐domain scatter correction for cone beam computed tomography using a residual convolutional neural network. Med Phys. 2019;46:3142–3155. 31077390 10.1002/mp.13583 PMC6684491

[93] Kurz C , Maspero M , Savenije MHF , et al. CBCT correction using a cycle‐consistent generative adversarial network and unpaired training to enable photon and proton dose calculation. Phys Med Biol. 2019;64:225004. 31610527 10.1088/1361-6560/ab4d8c

[94] Hansen DC , Landry G , Kamp F , et al. ScatterNet: a convolutional neural network for cone‐beam CT intensity correction. Med Phys. 2018;45:4916–4926. 30199101 10.1002/mp.13175

[95] Landry G , Hansen D , Kamp F , et al. Comparing Unet training with three different datasets to correct CBCT images for prostate radiotherapy dose calculations. Phys Med Biol. 2019;64:035011. 30523998 10.1088/1361-6560/aaf496

[96] Adrian T , Paolo Z , Arturs M , et al. Comparison of CBCT based synthetic CT methods suitable for proton dose calculations in adaptive proton therapy. Phys Med Biol. 2020;65:095002. 32143207 10.1088/1361-6560/ab7d54

[97] Kida S , Nakamoto T , Nakano M , et al. Cone beam computed tomography image quality improvement using a deep convolutional neural network. Cureus. 2018;10:e2548. 29963342 10.7759/cureus.2548 PMC6021187

[98] Xie S , Yang C , Zhang Z , Li H . Scatter artifacts removal using learning‐based method for CBCT in IGRT system. IEEE Access. 2018;6:78031–78037.

[99] Hwang D , Kang SK , Kim KY , et al. Generation of PET attenuation map for whole‐body time‐of‐flight 18F‐FDG PET/MRI using a deep neural network trained with simultaneously reconstructed activity and attenuation maps. J Nucl Med. 2019;60:1183–1189. 30683763 10.2967/jnumed.118.219493 PMC6681691

[100] Brenner DJ , Hall EJ . Computed tomography — an increasing source of radiation exposure. N Engl J Med. 2007;357:2277–2284. 18046031 10.1056/NEJMra072149

[101] Lin EC . Radiation risk from medical imaging. Mayo Clin Proc. 2010;85:1142–1146. 21123642 10.4065/mcp.2010.0260 PMC2996147

[102] Nguyen PK , Wu JC . Radiation exposure from imaging tests: is there an increased cancer risk? Expert Rev Cardiovasc Ther. 2011;9:177–183. 21453214 10.1586/erc.10.184 PMC3102578

[103] Brenner D , Elliston C , Hall E , Berdon W . Estimated risks of radiation‐induced fatal cancer from pediatric CT. Am J Roentgenol. 2001;176:289–296. 11159059 10.2214/ajr.176.2.1760289

[104] Lu B , Lu H , Palta J . A comprehensive study on decreasing the kilovoltage cone‐beam CT dose by reducing the projection number. J Appl Clin Med Phys. 2010;11:3274. 20717096 10.1120/jacmp.v11i3.3274 PMC5720437

[105] Tian Z , Jia X , Yuan K , Pan T , Jiang SB . Low‐dose CT reconstruction via edge‐preserving total variation regularization. Phys Med Biol. 2011;56:5949–5967. 21860076 10.1088/0031-9155/56/18/011 PMC4026331

[106] Wang T , Zhu L . Dual energy CT with one full scan and a second sparse‐view scan using structure preserving iterative reconstruction (SPIR). Phys Med Biol. 2016;61:6684. 27552793 10.1088/0031-9155/61/18/6684 PMC6200581

[107] Wang T , Zhu L . Pixel‐wise estimation of noise statistics on iterative CT reconstruction from a single scan. Med Phys. 2017;44:3525–3533. 28444799 10.1002/mp.12302

[108] Kalender WA , Wolf H , Suess C . Dose reduction in CT by anatomically adapted tube current modulation. II. Phantom measurements. Med Phys. 1999;26:2248–2253. 10587205 10.1118/1.598738

[109] Yu L , Liu X , Leng S , et al. Radiation dose reduction in computed tomography: techniques and future perspective. Imaging Med. 2009;1:65–84. 22308169 10.2217/iim.09.5 PMC3271708

[110] Padole A , Ali Khawaja RD , Kalra MK , Singh S . CT radiation dose and iterative reconstruction techniques. Am J Roentgenol. 2015;204:W384–W392. 25794087 10.2214/AJR.14.13241

[111] Jia X , Dong B , Lou Y , Jiang SB . GPU‐based iterative cone‐beam CT reconstruction using tight frame regularization. Phys Med Biol. 2011;56:3787–3807. 21628778 10.1088/0031-9155/56/13/004

[112] Niu T , Zhu L . Accelerated barrier optimization compressed sensing (ABOCS) reconstruction for cone‐beam CT: phantom studies. Med Phys. 2012;39:4588–4598. 22830790 10.1118/1.4729837 PMC3412436

[113] Zhang R , Thibault JB , Bouman CA , Sauer KD , Hsieh J . Model‐based iterative reconstruction for dual‐energy X‐Ray CT using a joint quadratic likelihood model. IEEE Trans Med Imaging. 2014;33:117–134. 24058024 10.1109/TMI.2013.2282370

[114] Jin P , Bouman CA , Sauer KD . A model‐based image reconstruction algorithm with simultaneous beam hardening correction for X‐ray CT. IEEE Trans Comput Imaging. 2015;1:200–216.

[115] Elbakri IA , Fessler JA . Statistical image reconstruction for polyenergetic X‐ray computed tomography. IEEE Trans Med Imaging. 2002;21:89–99. 11929108 10.1109/42.993128

[116] Zhao W , Niu K , Schafer S , Royalty K . An indirect transmission measurement‐based spectrum estimation method for computed tomography. Phys Med Biol. 2014;60:339–357. 25503491 10.1088/0031-9155/60/1/339

[117] Harms J , Wang T , Petrongolo M , Zhu L . Noise suppression for energy‐resolved CT using similarity‐based non‐local filtration. Paper presented at: SPIE Medical Imaging; 2016;9783:8.

[118] Harms J , Wang T , Petrongolo M , Niu T , Zhu L . Noise suppression for dual‐energy CT via penalized weighted least‐square optimization with similarity‐based regularization. Med Phys. 2016;43:2676–2686. 27147376 10.1118/1.4947485 PMC4859835

[119] Dong J , Fu J , He Z . A deep learning reconstruction framework for X‐ray computed tomography with incomplete data. PLoS One. 2019;14:e0224426. 31675363 10.1371/journal.pone.0224426 PMC6824569

[120] Jin KH , McCann MT , Froustey E , Unser M . Deep convolutional neural network for inverse problems in imaging. IEEE Trans Image Process. 2017;26:4509–4522. 28641250 10.1109/TIP.2017.2713099

[121] Shan H , Padole A , Homayounieh F , et al. Competitive performance of a modularized deep neural network compared to commercial algorithms for low‐dose CT image reconstruction. Nature Machine Intelligence. 2019;1:269–276. 10.1038/s42256-019-0057-9 PMC7687920 33244514

[122] Kang E , Min J , Ye JC . A deep convolutional neural network using directional wavelets for low‐dose X‐ray CT reconstruction. Med Phys. 2017;44:e360–e375. 29027238 10.1002/mp.12344

[123] McCollough CH , Bartley AC , Carter RE , et al. Low‐dose CT for the detection and classification of metastatic liver lesions: results of the 2016 low dose CT grand challenge. Med Phys. 2017;44:e339–e352. 29027235 10.1002/mp.12345 PMC5656004

[124] Yi X , Babyn P . Sharpness‐aware low‐dose CT denoising using conditional generative adversarial network. J Digit Imaging. 2018;31:655–669. 29464432 10.1007/s10278-018-0056-0 PMC6148809

[125] Lei Y , Fu Y , Mao H , Curran W , Liu T , Yang X . Multi‐modality MRI arbitrary transformation using unified generative adversarial networks. Paper presented at: SPIE Medical Imaging; 2020:11313.

[126] Qu L , Zhang Y , Wang S , Yap P‐T , Shen D . Synthesized 7T MRI from 3T MRI via deep learning in spatial and wavelet domains. Med Image Anal. 2020;62:101663. 32120269 10.1016/j.media.2020.101663 PMC7237331

[127] Schlemper J , Caballero J , Hajnal JV , Price AN , Rueckert D . A deep cascade of convolutional neural networks for dynamic MR image reconstruction. IEEE Trans Med Imaging. 2018;37:491–503. 29035212 10.1109/TMI.2017.2760978

[128] Shiri I , Ghafarian P , Geramifar P , et al. Direct attenuation correction of brain PET images using only emission data via a deep convolutional encoder‐decoder (Deep‐DAC). Eur Radiol. 2019;29:6867–6879. 31227879 10.1007/s00330-019-06229-1

[129] An L , Zhang P , Adeli E , et al. Multi‐level canonical correlation analysis for standard‐dose PET image estimation. IEEE Trans Image Process. 2016;25:3303–3315. 27187957 10.1109/TIP.2016.2567072 PMC5106345

[130] Sanaat A , Arabi H , Mainta I , Garibotto V , Zaidi H . Projection‐space implementation of deep learning‐guided low‐dose brain PET imaging improves performance over implementation in image‐space. J Nucl Med. 2020;61:1388–1396. 31924718 10.2967/jnumed.119.239327 PMC7456177

[131] Chen KT , Gong E , de Carvalho Macruz FB , et al. Ultra‐low‐dose (18)F‐florbetaben amyloid pet imaging using deep learning with multi‐contrast MRI inputs. Radiology. 2019;290:649–656. 30526350 10.1148/radiol.2018180940 PMC6394782

[132] Xiang L , Qiao Y , Nie D , An L , Wang Q , Shen D . Deep auto‐context convolutional neural networks for standard‐dose PET image estimation from low‐dose PET/MRI. Neurocomputing. 2017;267:406–416. 29217875 10.1016/j.neucom.2017.06.048 PMC5714510

[133] Schilling KG , Blaber J , Huo Y , et al. Synthesized b0 for diffusion distortion correction (Synb0‐DisCo). Magn Reson Imaging. 2019;64:62–70. 31075422 10.1016/j.mri.2019.05.008 PMC6834894

[134] Azizi S , Mousavi P , Yan P , et al. Transfer learning from RF to B‐mode temporal enhanced ultrasound features for prostate cancer detection. Int J Comput Assist Radiol Surg. 2017;12:1111–1121 28349507 10.1007/s11548-017-1573-x PMC8171585

[135] Gulshan V , Peng L , Coram M , et al. Development and validation of a deep learning algorithm for detection of diabetic retinopathy in retinal fundus photographs. JAMA. 2016;316:2402–2410. 27898976 10.1001/jama.2016.17216

[136] Mohamed AA , Berg WA , Peng H , Luo Y , Jankowitz RC , Wu S . A deep learning method for classifying mammographic breast density categories. Med Phys. 2018;45:314–321. 29159811 10.1002/mp.12683 PMC5774233

[137] Sun C , Shrivastava A , Singh S , Gupta A . Revisiting Unreasonable Effectiveness of Data in Deep Learning Era; 2017arXiv:1707.02968. https://ui.adsabs.harvard.edu/abs/2017arXiv170702968S Accessed July 01, 2017.

[138] Shin H , Roth HR , Gao M et al Deep convolutional neural networks for computer‐aided detection: CNN architectures, dataset characteristics and transfer learning. IEEE Trans Med Imaging. 2016;35:1285–1298. 26886976 10.1109/TMI.2016.2528162 PMC4890616

[139] Chen H , Gu J , Gallo O , Liu M , Veeraraghavan A , Kautz J . Reblur2Deblur: Deblurring videos via self‐supervised learning. Paper presented at: 2018 IEEE International Conference on Computational Photography (ICCP); 4–6 May 2018, 2018 10.1109/ICCPHOT.2018.8368468:1-9

[140] Ahn J , Kwak S . Learning Pixel‐level Semantic Affinity with Image‐level Supervision for Weakly Supervised Semantic Segmentation. 2018.arXiv:1803.10464. https://ui.adsabs.harvard.edu/abs/2018arXiv180310464A Accessed March 01, 2018.

[141] Shin H‐C , Tenenholtz NA , Rogers JK , et al. Medical Image Synthesis for Data Augmentation and Anonymization Using Generative Adversarial Networks; 2018.1‐11; Cham.

[142] Brou Boni KND , Klein J , Vanquin L , et al. MR to CT synthesis with multicenter data in the pelvic area using a conditional generative adversarial network. Phys Med Biol. 2020;65:075002. 32053808 10.1088/1361-6560/ab7633

[143] Xiang L , Wang Q , Nie D , et al. Deep embedding convolutional neural network for synthesizing CT image from T1‐weighted MR image. Med Image Anal. 2018;47:31–44. 29674235 10.1016/j.media.2018.03.011 PMC6410565

[144] Maspero M , Savenije MHF , Dinkla AM , et al. Dose evaluation of fast synthetic‐CT generation using a generative adversarial network for general pelvis MR‐only radiotherapy. Phys Med Biol. 2018;63:185001. 30109989 10.1088/1361-6560/aada6d

[145] Liu F , Yadav P , Baschnagel AM , McMillan AB . MR‐based treatment planning in radiation therapy using a deep learning approach. J Appl Clin Med Phys. 2019;20:105–114. 10.1002/acm2.12554 PMC6414148 30861275

[146] Wang Y , Liu C , Zhang X , Deng W . Synthetic CT generation based on T2 weighted MRI of nasopharyngeal carcinoma (NPC) using a deep convolutional neural network (DCNN). Front Oncol. 2019;9:1333. 31850218 10.3389/fonc.2019.01333 PMC6901977

[147] Koike Y , Akino Y , Sumida I , et al. Feasibility of synthetic computed tomography generated with an adversarial network for multi‐sequence magnetic resonance‐based brain radiotherapy. J Radiat Res. 2020;61:92–103. 31822894 10.1093/jrr/rrz063 PMC6976735

[148] Jin C‐B , Kim H , Liu M , et al. DC2Anet: generating lumbar spine MR images from CT scan data based on semi‐supervised learning. Appl Sci. 2019;9:2521.

[149] Jin C‐B , Kim H , Liu M , et al. Deep CT to MR Synthesis Using Paired And Unpaired Data. Sensors. 2019;19:2361. 10.3390/s19102361 PMC6566351 31121961

[150] Lee JH , Han IH , Kim DH , et al. Spine computed tomography to magnetic resonance image synthesis using generative adversarial networks: a preliminary study. J Korean Neurosurg Soc. 2020;63:386–396. 31931556 10.3340/jkns.2019.0084 PMC7218205

[151] Li Y , Zhu J , Liu Z , et al. A preliminary study of using a deep convolution neural network to generate synthesized CT images based on CBCT for adaptive radiotherapy of nasopharyngeal carcinoma. Phys Med Biol. 2019;64:145010. 31170699 10.1088/1361-6560/ab2770

[152] Yuan N , Dyer B , Rao S , et al. Convolutional neural network enhancement of fast‐scan low‐dose cone‐beam CT images for head and neck radiotherapy. Phys Med Biol. 2020;65:035003. 31842014 10.1088/1361-6560/ab6240 PMC8011532

[153] Kida S , Kaji S , Nawa K , et al. Visual enhancement of cone‐beam CT by use of CycleGAN. Med Phys. 2020;47:998–1010. 31840269 10.1002/mp.13963

[154] Liu F , Jang H , Kijowski R , Zhao G , Bradshaw T , McMillan AB . A deep learning approach for (18)F‐FDG PET attenuation correction. EJNMMI Phys. 2018;5:24. 30417316 10.1186/s40658-018-0225-8 PMC6230542

[155] Armanious K , Kustner T , Reimold M , et al. Independent brain (18)F‐FDG PET attenuation correction using a deep learning approach with generative adversarial networks. Hell J Nucl Med. 2019;22:179–186. 31587027 10.1967/s002449911053

[156] Chen H , Zhang Y , Kalra MK , et al. Low‐dose CT with a residual encoder‐decoder convolutional neural network. IEEE Trans Med Imaging. 2017;36:2524–2535. 28622671 10.1109/TMI.2017.2715284 PMC5727581

[157] Wolterink JM , Leiner T , Viergever MA , Išgum I . Generative adversarial networks for noise reduction in low‐dose CT. IEEE Trans Med Imaging. 2017;36:2536–2545. 28574346 10.1109/TMI.2017.2708987

[158] Yang W , Zhang H , Yang J , et al. Improving low‐dose CT image using residual convolutional network. IEEE Access. 2017;5:24698–24705.

[159] Kang E , Chang W , Yoo J , Ye JC . Deep convolutional framelet denosing for low‐dose CT via wavelet residual network. IEEE Trans Med Imaging. 2018;37:1358–1369. 29870365 10.1109/TMI.2018.2823756

[160] Shan H , Zhang Y , Yang Q , et al. 3‐D convolutional encoder‐decoder network for low‐dose CT via transfer learning from a 2‐D trained network. IEEE Trans Med Imaging. 2018;37:1522–1534. 29870379 10.1109/TMI.2018.2832217 PMC6022756

[161] You C , Yang Q , Shan H , et al. Structurally‐sensitive multi‐scale deep neural network for low‐dose CT denoising. IEEE Access. 2018;6:41839–41855. 30906683 10.1109/ACCESS.2018.2858196 PMC6426337

[162] Han Y , Ye JC . Framing U‐Net via deep convolutional framelets: application to sparse‐view CT. IEEE Trans Med Imaging. 2018;37:1418–1429. 29870370 10.1109/TMI.2018.2823768

[163] Liu Y , Zhang Y . Low‐dose CT restoration via stacked sparse denoising autoencoders. Neurocomputing. 2018;284:80–89.

[164] Zhao T , McNitt‐Gray M , Ruan D . A convolutional neural network for ultra‐low‐dose CT denoising and emphysema screening. Med Phys. 2019;46:3941–3950. 31220358 10.1002/mp.13666

[165] Lee H , Lee J , Kim H , Cho B , Cho S . Deep‐neural‐network‐based sinogram synthesis for sparse‐view CT image reconstruction. IEEE Trans Radiat Plasma Med Sci. 2019;3:109–119.

[166] Gong E , Pauly JM , Wintermark M , Zaharchuk G . Deep learning enables reduced gadolinium dose for contrast‐enhanced brain MRI. J Magn Reson Imaging. 2018;48:330–340. 29437269 10.1002/jmri.25970

[167] Chartsias A , Joyce T , Giuffrida MV , Tsaftaris SA . Multimodal MR synthesis via modality‐invariant latent representation. IEEE Trans Med Imaging. 2018;37:803–814. 29053447 10.1109/TMI.2017.2764326 PMC5904017

[168] Quan TM , Nguyen‐Duc T , Jeong W . Compressed sensing MRI reconstruction using a generative adversarial network with a cyclic loss. IEEE Trans Med Imaging. 2018;37:1488–1497. 29870376 10.1109/TMI.2018.2820120

[169] Galbusera F , Bassani T , Casaroli G , et al. Generative models: an upcoming innovation in musculoskeletal radiology? A preliminary test in spine imaging. Eur Radiol Experim. 2018;2:29. 10.1186/s41747-018-0060-7 PMC6207611 30377873

[170] Dar SU , Yurt M , Karacan L , Erdem A , Erdem E , Çukur T . Image synthesis in multi‐contrast MRI with conditional generative adversarial networks. IEEE Trans Med Imaging. 2019;38:2375–2388. 30835216 10.1109/TMI.2019.2901750

[171] Mardani M , Gong E , Cheng JY , et al. Deep Generative Adversarial Neural networks for compressive sensing MRI. IEEE Trans Med Imaging. 2019;38:167–179. 30040634 10.1109/TMI.2018.2858752 PMC6542360

[172] Wei W , Poirion E , Bodini B , et al. Fluid‐attenuated inversion recovery MRI synthesis from multisequence MRI using three‐dimensional fully convolutional networks for multiple sclerosis. Journal of Medical Imaging. 2019;6:014005. 30820439 10.1117/1.JMI.6.1.014005 PMC6379787

[173] Huang W , Luo M , Liu X , et al. Arterial spin labeling images synthesis from sMRI using unbalanced deep discriminant learning. IEEE Trans Med Imaging. 2019;38:2338–2351. 30908201 10.1109/TMI.2019.2906677

[174] Liu F , Feng L , Kijowski R . MANTIS: model‐augmented neural network with incoherent k‐space sampling for efficient MR parameter mapping. Magn Reson Med. 2019;82:174–188. 30860285 10.1002/mrm.27707 PMC7144418

[175] Wu Y , Ma Y , Capaldi DP , et al. Incorporating prior knowledge via volumetric deep residual network to optimize the reconstruction of sparsely sampled MRI. Magn Reson Imaging. 2020;66:93–103. 30880112 10.1016/j.mri.2019.03.012 PMC6745016

[176] Fujita S , Hagiwara A , Otsuka Y , et al. Deep learning approach for generating MRA images from 3D quantitative synthetic MRI without additional scans. Invest Radiol. 2020;55:249–256. 31977603 10.1097/RLI.0000000000000628

[177] Yang J , Park D , Gullberg GT , Seo Y . Joint correction of attenuation and scatter in image space using deep convolutional neural networks for dedicated brain (18)F‐FDG PET. Phys Med Biol. 2019;64:075019. 30743246 10.1088/1361-6560/ab0606 PMC6449185

[178] Wang Y , Yu B , Wang L , et al. 3D conditional generative adversarial networks for high‐quality PET image estimation at low dose. NeuroImage. 2018;174:550–562. 29571715 10.1016/j.neuroimage.2018.03.045 PMC6410574

[179] Gong K , Guan J , Liu C , Qi J . PET image denoising using a deep neural network through fine tuning. IEEE Trans Radiat Plasma Med Sci. 2019;3:153–161. 32754674 10.1109/TRPMS.2018.2877644 PMC7402614

[180] Haggstrom I , Schmidtlein CR , Campanella G , DeepPET FTJ . A deep encoder‐decoder network for directly solving the PET image reconstruction inverse problem. Med Image Anal. 2019;54:253–262. 30954852 10.1016/j.media.2019.03.013 PMC6537887

[181] Wang Y , Zhou L , Yu B et al 3D auto‐context‐based locality adaptive multi‐modality GANs for PET synthesis. IEEE Trans Med Imaging. 2019;38:1328–1339. 30507527 10.1109/TMI.2018.2884053 PMC6541547

[182] Lu W , Onofrey JA , Lu Y , et al. An investigation of quantitative accuracy for deep learning based denoising in oncological PET. Phys Med Biol. 2019;64:165019. 31307019 10.1088/1361-6560/ab3242

[183] Kaplan S , Zhu Y‐M . Full‐dose PET image estimation from low‐dose PET image using deep learning: a pilot study. J Digit Imaging. 2019;32:773–778. 30402670 10.1007/s10278-018-0150-3 PMC6737135

